# Discounting of Future Rewards and Punishments in Rats

**DOI:** 10.1523/ENEURO.0452-21.2022

**Published:** 2022-11-30

**Authors:** Maurice-Philipp Zech, Sandra Schäble, Tobias Kalenscher

**Affiliations:** Comparative Psychology, Institute of Experimental Psychology, Heinrich-Heine Universität Düsseldorf, Düsseldorf, 40225, Germany

**Keywords:** aversive discounting, delay discounting, intertemporal choice, reward, shock, utility from anticipation

## Abstract

Temporal reward discounting describes the decrease of value of a reward as a function of delay. Decision-making between future aversive outcomes is much less studied, and there is no clear decision pattern across studies; while some authors suggest that human and nonhuman animals prefer sooner over later painful shocks, others found the exact opposite. In a series of three experiments, Long–Evans rats chose between differently timed electric shocks and rewards in a T-maze. In experiment 1, rats chose between early and late painful shocks with identical, long reward delays; in experiment 2, they chose between early reward and early shocks, or late rewards and late shocks; in experiment 3, they chose between early and late rewards, with identical, short delays to the shock. We tested the predictions of two competing hypotheses: the aversive discounting theory assumes that future shocks are discounted, and, hence, less unpleasant than early shocks. The utility from anticipation theory implies that rats derive negative utility from waiting for the shock; late shocks should, hence, be more unpleasant than early shocks. We did not find unanimous evidence for either theory. Instead, our results are more consistent with the *post hoc* idea that shocks may have negative spill-over effects on reward values, the closer in time a shock is to a subsequent reward, the stronger the reward is devalued. Interestingly and consistent with our theory, we find that, depending on the temporal shock-reward contiguity, rats can be brought to prefer later over sooner rewards of identical magnitudes.

## Significance Statement

It is well understood how animals discount future rewards, but much less is known about how they choose between future aversive outcomes. We designed a novel task to examine decision-making between differently timed electric shocks and rewards. Although rats revealed clear preferences for sooner over later shocks, we found no evidence that they derived negative utility from waiting for the shocks (dread), nor that they discounted the disutility of future shocks. Instead, their choices were consistent with the novel hypothesis that shocks have negative, time-dependent spill-over effects on the utility of subsequent rewards. Consistent with this, we find that, depending on the temporal shock-reward contiguity, rats can be brought to prefer later over sooner rewards, thus promoting self-control.

## Introduction

Individuals devalue rewards as a function of time until they can be realized, a phenomenon called temporal discounting (Kalenscher and Pennartz, 2008; Woolverton et al., 2012). Not only humans, but also nonhuman, animals, including rats, mice, and pigeons, discount future rewards (Kalenscher et al., 2005; Vanderveldt et al., 2016). However, although temporal reward discounting is reasonably well understood, most real-life decisions often also yield negative consequences in the future, e.g., negative health effects of smoking, or diet-related health problems, a phenomenon less well studied. Several competing theories have been developed to account for decisions between delayed aversive events.

The aversive discounting model assumes a decrease of aversiveness as a function of delay. That is, a future event with negative utility would be less unpleasant than the same event now. This theory was empirically supported by several studies in rats who preferred later over earlier electric shocks (Deluty, 1978; Liley et al., 2019). Another study provided further support that the aversiveness of unpleasant events decreases with increasing delay (Woolverton et al., 2012). Interestingly, the decline of the negative value of the aversive event was best descripted by a hyperbolic function similar to temporal reward discounting.

However, contrary to the predictions of the aversive discounting model, many individuals tend to accelerate, rather than defer, aversive future events. For instance, many people tend to choose earlier over later painful dentist appointments, although the aversive discounting model would predict that the later appointment should be less frightening. The utility from anticipation model (Loewenstein, 1987) can account for this behavior. It states that the final utility of a delayed aversive event is the result of two interacting mental processes: the discounting of the aversiveness of a future aversive event, as hypothesized by the aversive discounting model, plus the disutility derived from anticipating the aversive event (dread). In other words, the final utility of a future outcome is a combination of the utility derived from anticipating the outcome and the discounted utility of future consumption. If the negative utility from anticipation outweighs the discounted negative utility from the event itself, people aspire to reduce the aversive anticipation period and thus accelerate the event (“get it over with”). Applied to the example above, the utility from anticipation model predicts that the dread of waiting for the painful dental procedure would motivate the acceleration of the appointment. Both the utility from anticipation model as well as the aversive discounting model make similar predictions regarding the discounting of future rewards.

In support of this model, human participants have been shown to not only accelerate electric shocks, they were even willing to endure a stronger shock to avoid waiting for it (Berns et al., 2006). In another study, human participants preferred a smaller, sooner over a larger, later monetary loss (Thaler and Shefrin, 1981; Holt et al., 2008). In general, humans discount delayed gains more steeply than delayed losses indicating different processes for the discounting of positive and negative outcomes (Estle et al., 2006; Mies et al., 2016). This effect is called the sign effect and participants exhibiting the sign effect show different neural activity compared with participants not experiencing the sign effect (Tanaka et al., 2014). Sign effects are observed for monetary losses, but also primary punishers like the threat of a shock (Robinson et al., 2015). Nonhuman animals also show behavior consistent with the utility from anticipation model. In one study, rats preferred an immediate electric shock over a delayed shock (Knapp et al., 1959). Additionally, Rodríguez et al. (2018) presented rats the choice between a large reward paired with a shock, and a small reward without shock. Without any delay between the large reward and the shock, the small reward was preferred. By increasing the delay between the large reward and the shock, rats chose the larger reward with shock. However, note that an alternative interpretation of the rats’ preference for early over late shocks is that the uncertainty of the timing of the shock increases with increasing delay. Animals may prefer sooner over later shocks to make more appropriate preparatory responses (Knapp et al., 1959; Seligman et al., 1971).

In summary, there is contradicting evidence in the human and nonhuman literature about decision-making about future aversive events: some evidence suggests that future aversive events are less unpleasant than immediate events, much like future rewards are less appetitive than immediate rewards. However, other studies imply that humans and nonhuman animals prefer earlier over later aversive events. In the human literature, this ambiguity has been resolved by assuming that primary punishment, such as a painful event, is more dreadful, and, hence, generates more disutility from anticipation, than secondary punishment, such as delayed financial payments (Loewenstein, 1987; Benzion et al., 1989). However, this idea cannot explain the contradicting evidence in the animal literature where primary reinforcement and punishment are used predominantly. Hence, it is unclear whether animals prefer an early aversive over a later event, as predicted by the utility from anticipation model, or vice versa, in line with the predictions of the aversive discounting model.

In the current study, we tested whether rats choose early or late electric shocks. Across repeated trials, they could enter one of two arms in a T-maze. Both arms yielded rewards and shocks, both delivered with variable delays. We tested the predictions of both theories in a series of three experiments by altering entry-to-shock delays and shock-to-reward delays. We additionally employed exploratory analyses.

## Materials and Methods

### Housing and animals

The rats for all experiments were obtained from Charles River Laboratories (Calco) and kept in an inverted 12/12 h light/dark cycle (light off at 7 A.M.). The temperature within the colony room was maintained at 20 ± 2°C and the humidity at 50%. Upon arrival the animals were between eight and nine weeks old, and the food access was set *ad libitum* until 3 days before the experiment. Subsequently, the food was restricted to maintain animals at >90% of their free-feeding body weight. Standard rodent laboratory food (Sniff) was used. Throughout the experiments, water access was *ad libitum*. We used 25 (experiment 1), 20 (experiment 2), and 21 (experiment 3) male Long–Evans rats. Rats were always housed in groups of three per cage (59 × 38 × 20 cm). The rats were weighed every day to monitor their health. All experiments were conducted according to the European Union Directive 2017/63/EU and approved by the German authorities (Landesamt für Natur, Umwelt und Verbraucherschutz, NRW).

### Experimental setup

**Figure 1.**
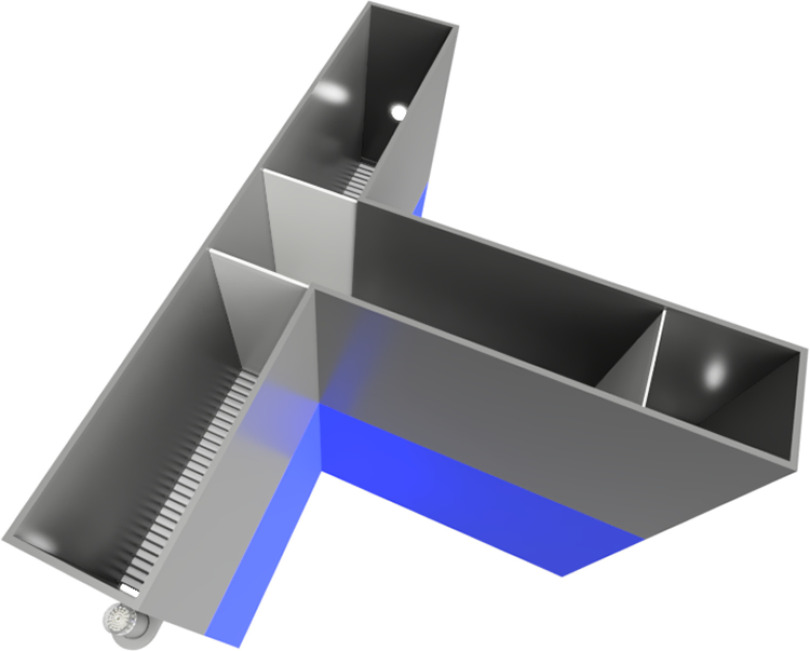
Overview of the customized T-maze. The start arm starts on the right side with the start box, which can be closed by an automatic door. Two identical decision arms were connected to the start arm. Additionally, the decision arms could be closed by automatic down sliding doors. Pellet dispensers were placed at the end of the start arm and each decision arm. Finally, the pellets were delivered into Petri dishes, and above the Petri dishes, reward lights were placed.

A customized T-maze was used in all experiments (Fig. 1). The T-maze consisted of a start arm (80 × 30 × 45 cm), including a start box (40 × 30 × 45 cm). The start arm was connected to two identical decisions arms (50 × 30 × 45 cm) leading left and right, respectively. The floor of the maze consisted of grid floors (9-mm gaps) to apply electric shocks. Each decision arm and the start box were separated by automatic sliding doors that could be lowered. Additionally, pellet dispensers were placed at the end of each decision arm and the start box. The pellet dispensers delivered the food rewards (20 mg, dustless precision pellets, Bio-Serv) into Petri dishes and reward lights were placed above the Petri dishes. The apparatus was controlled by Ethovision 11.5 (Noldus Information Technology).

### Shaping and pretraining

The general procedure of shaping and pretraining of all studies was identical. Training and testing were done in the active phase of the animals and only on weekdays. The subjects were habituated to the maze for 1 day (see the Appendix for the details on the habituation and shaping procedures). Subsequently, rats were trained for four sessions in shaping 1. Next was five sessions of shaping 2, followed by 3 d of shaping 3. Independent of the performance of the animals all training phases were conducted. After the last step a performance criterion was applied to determine which animals were promoted to the main experiment consisting of 10 sessions. Food rewards always consisted of three sucrose pellets (20 mg, dustless precision pellets, Bio-Serv). The reward lights signaled the availability of food rewards.

### Hypotheses

**Figure 2.**
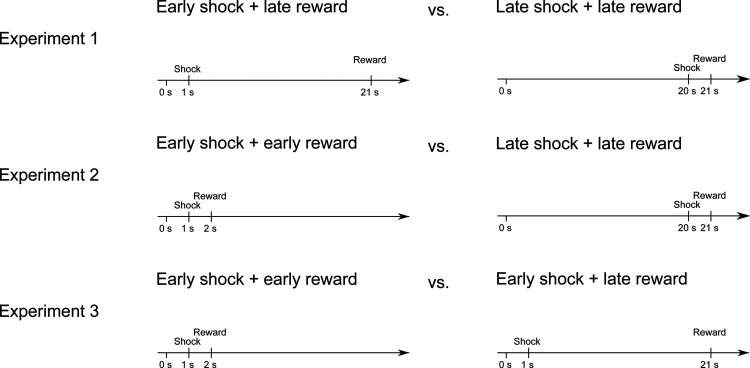
Overview of the shock and reward timings for each experiment. After an animal entered a decision arm (0 s), the doors were closed. Afterwards, the rewards and shocks were delivered. In general, an early reward had a delay of 2 s and a late reward of 21 s. The early shock was administered after 1 s (relative to entering) and the late shock after 20 s.

We tested rats’ preferences between timed rewards and shocks in three experiments. In experiment 1, the underlying logic was that rats choose between alternatives that yield identical rewards, delivered after identical delays, but differ with respect to the timing of the shock. Rats entered each decision arm in the T-maze and received a reward 21 s after arm-entry (Fig. 2). Entering one decision arm yielded a shock after 1 s (early shock + late reward; EL), entering the other arm yielded a shock after 20 s (late shock + late reward; LL). The aversive discounting theory predicts that rats would choose the arm yielding a late shock because the negative value of late shocks should be discounted; late shocks should, thus, be less aversive than early shocks at the time point of decision. By contrast, the utility from anticipation theory would predict choices of the early shock because of increasing dread with longer delays.

**Figure 3.**
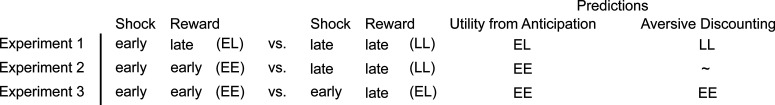
Overview of the shock and reward contingencies in the two arms of the T-maze for each experiment and the predictions for each theory. For the shocks and rewards, each column represents one arm of the T-maze (early reward + late reward [EE]; late shock + late reward [LL]; early shock + early reward [EE]). In the first experiment, the entry-to-reward contingencies are identical in both arms, but the entry-to-shock delays differ between both arms. The second experiment tested constant shock-to-reward delays with different entry-to-shock delays. The third experiment used constant entry-to-shock delays, but the shock-to-reward contingencies differ between arms. In the predictions, “∼” represents no predictions. Note that the assignment of shock/reward contingencies to the left or right arm of the T-maze will be pseudo-randomized across sessions for all experiments. The left side is always the condition for which the percentage of decisions was calculated.

Note that this experimental design confounds entry-to-shock delay (the delay between entering the arm and receiving the shock) with shock-to-reward delay (late shocks are closer in time to reward than early shocks). A choice of late rewards could be indicative of utility from anticipation, as outlined above, but, given this confound, it is equally plausible that rats might use the shock as a cue to predict the following reward; hence, if this was true, the delay between cue and reward would be shorter in LL trials, temporal reward discounting would therefore predict choices of the late shock. Therefore, in experiment 2, we kept the shock-to-reward delay constant across choices. Choices of one arm yielded an early shock 1 s after entering, followed by an early reward 1 s after the shock (EE), choices of the other arm yielded a late shock, followed by a late reward (LL; same timings as above). The utility from anticipation theory would predict choices of the early shock/early reward arm to minimize delay-to-shock (dread) and delay-to-reward (sooner rewards are better than later rewards). The predictions of the aversive discounting model are somewhat unclear since the discounted disutility of the late shock (late shocks are better than early shocks) would compete with the discounted utility of the late reward (late rewards are worse than early rewards). Either way, any choice could not be accounted for by shock-to-reward signaling. To tease out the role of the discounted reward in experiment 2, we manipulated the entry-to-reward delay in experiment 3. Rats chose between identically timed, early shocks (1-s latency), and an early (EE, 2-s latency) or a late reward (EL; 21-s latency). Thus, standard temporal reward discounting would predict choices of the earlier over the later reward. Figure 3 summarizes the shock and reward contingencies and theory predictions. The following paragraph provides further details of the experimental designs.

### Experimental sessions

We used a between-subject design, each experiment was performed by a separate batch of rats. In each experiment, rats performed 10 sessions (one session per day), consisting of 21 trials. Each session began with six forced trials in which only one arm was opened to ensure the rats were sampling both decision arms with their respective, entry-to-reward as well as shock-to-reward delays. Upon completion of the forced trials, the animals performed 16 free trials in which they could choose between both arms. Importantly, the arm-outcome (shock/reward) contingencies were counterbalanced and pseudo-randomized within and across animals and across sessions; they, hence, had to be re-learned in each session. If the arm-outcome contingency was unchanged for more than two consecutive sessions, it was reversed in the subsequent session. Before and after each animal training, the maze was cleaned with a 70% ethyl alcohol solution to remove odor cues.

### Typical trial structure

The animals always started in the start box indicating the start of one trial. Before all doors were opened (free trials), the reward light of the start box was illuminated and a food reward was delivered. Once an animal entered a decision arm all doors were closed and depending on the condition a specific shock-reward timing was used. Afterwards, the corresponding door of the decision arm and the door of the start box were opened. Upon entering the start arm, the door of the decision arm was closed. In case an animal did not stay in the decision arm, to avoid the shock, the trial was labeled as an omission trial. In omission trials no rewards were delivered and the reward light in the start box blinked three times. Finally, after entering the start box, the last door was closed and a new trial started. The animal was removed after completing all trials or after a duration of 40 min.

### Analysis

In all three experiments, animals performed nearly all trials (experiment 1: 99.20%; experiment 2: 99.97%; experiment 3: 99.85%). Additionally, trials in which animals avoided the shock were labeled as omission trials. However, because of the low occurrence rate (experiment 1: 0.80%; experiment 2: 0.02%; experiment 3: 0.13%), we excluded them from analysis. To test whether rats prefer one arm or the other in each experiment, we calculated one-sample *t* tests (Extended Data 1; two-tailed; Table 1), one for each experiment, against the 50% chance level with the dependent variable percentage of choices of each arm. To test for learning effects, for each experiment, we ran a repeated-measures ANOVA (Table 1) of the effect of trial block (block 1: trials 1–8 vs block 2: trials 9–16) and session number on the percentage of choice. A second repeated-measures ANOVA (within-subject-factors: first vs second block of sessions, i.e., block 1: sessions 1–5 vs block 2: sessions 6–10; session order within each block) was calculated to check whether the behavior changed over time. For the null hypothesis significance testing IBM SPSS Statistics 27 (IBM) and MATLAB 2019a (The MathWorks) were used. The level of significance for all statistical tests was α = 0.05.

In addition, we conducted Bayesian inference statistics and calculated the Bayesian posterior distribution for each experiment (Extended Data 1). A Bayesian framework of inference allowed us to calculate the highest density interval (HDI). Thus, assumptions can be made that a specific value is within the 95% most probable data (Wagenmakers et al., 2018).

For the Bayesian parameter estimation RStudio (RStudio Team, 2018) was used. Additionally, the following R packages were used: rstan (Stan Development Team, 2020) and patchwork (Pedersen, 2020).

**Figure 4.**
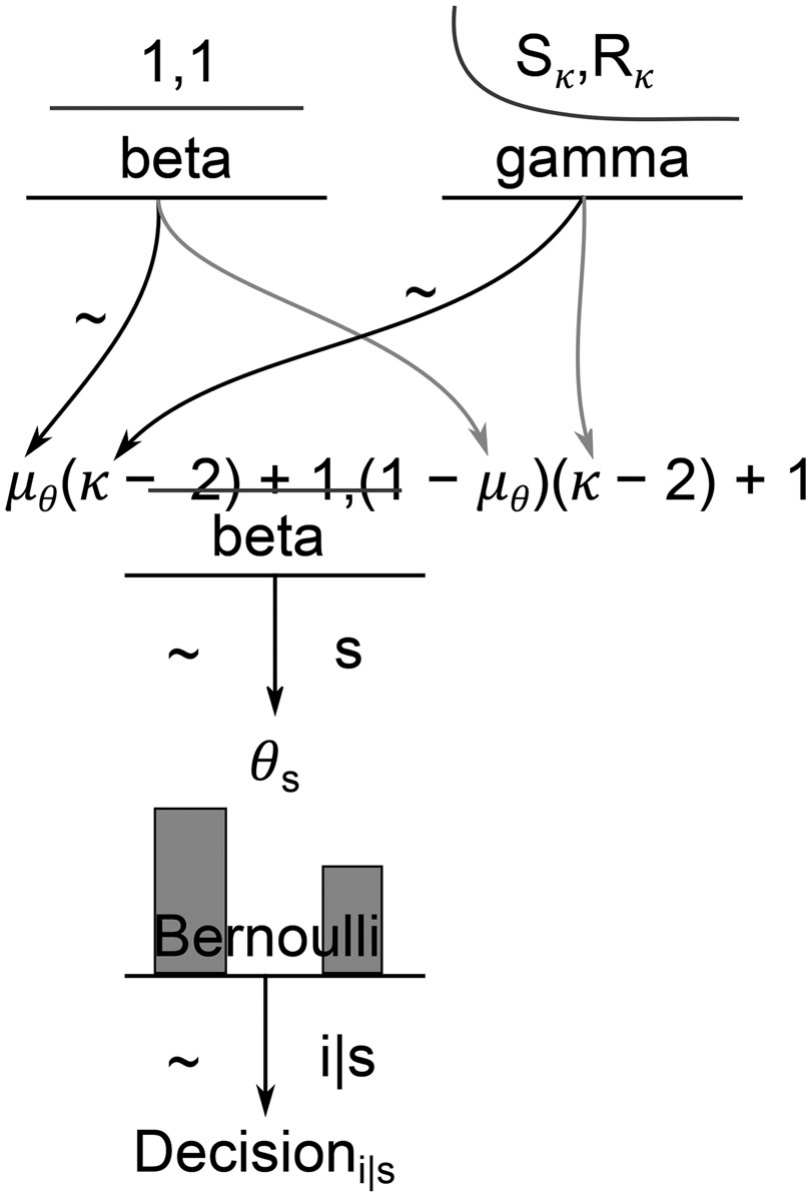
Depicted is the used Bayesian hierarchy model. As the likelihood function for the decisions, a Bernoulli distribution was used. Each estimation of θ followed a β function with $\mu_{\theta }\left(\kappa -2\right)+1,\left(1-\mu_{\theta }\right)\left(\kappa -2\right)+1$. For $\mu_{\theta }$, an uninformed β prior was used, and κ used a gamma(0.01,0.01) function (for prior predictions, see Extended Data Figs. 4-1, 4-2, 4-3).

We calculated the following model (Fig. 4) for each experiment with the general form of $Decision_{i|s}∼Bernoulli(\theta_{s})$ with $Decision_{i|s}\in {\mathbb{N}}_{\text{[}0\text{|}1]}and\theta_{s}\in {\mathbb{R}}_{\left[0,1\right]}$. $Decision_{i|s}$ is the $i_{th}$ Decision for $s_{th}$ subject and $\theta_{s}$ is the parameter estimate for the $s_{th}$ subject. We assume for $\theta_{s}∼beta(\mu_{\theta }\left(\kappa -2\right)+1,\left(1-\mu_{\theta }\right)\left(\kappa -2\right)+1)$ where $\mu_{\theta }\epsilon {\mathbb{R}}_{\left[0,1\right]}$ and $\kappa \in {\mathbb{R}}_{\geq 2}$. The mean parameter estimate for the group level is $\mu_{\theta }$ and κ is a parameter indicating whether $\theta_{s}$ is similar to $\mu_{\theta }$. Additionally, κ was modulated by κ∼ gamma(0.01,0.01). Hence, κ is estimated conservatively, i.e., the estimation of $\theta_{s}$ does not dependent on $\mu_{\theta }$. Finally, an uninformed prior was used with $\mu_{\theta }∼beta(1,1)$. In a nutshell, $\theta_{s}$ represents the mean parameter estimate for each animal and $\mu_{\theta }$ represents the mean parameter estimate on a group level.

10.1523/ENEURO.0452-21.2022.f4-1 Extended Data Figure 4-1 Prior distribution for the κ parameter. The *y*-axis represents the κ density and the x-axis the κ parameter. Download Figure 4-1, TIF file.

10.1523/ENEURO.0452-21.2022.f4-2 Extended Data Figure 4-2 Prior distribution for the omega parameter. The *y*-axis represents the omega density and the *x*-axis the omega parameter. Download Figure 4-2, TIF file.

10.1523/ENEURO.0452-21.2022.f4-3 Extended Data Figure 4-3 Prior distribution for the θ parameter. The *y*-axis represents the θ density and the *x*-axis the θ parameter. Download Figure 4-3, TIF file.

10.1523/ENEURO.0452-21.2022.ed1 Extended Data 1 Data and scripts for all calculations. Folders are separated for the data extraction (Data), null hypothesis testing (NHST), and bayesian hierarchical models (Bayes). Download Extended Data 1, ZIP file

### Simulated predictions

**Figure 5.**
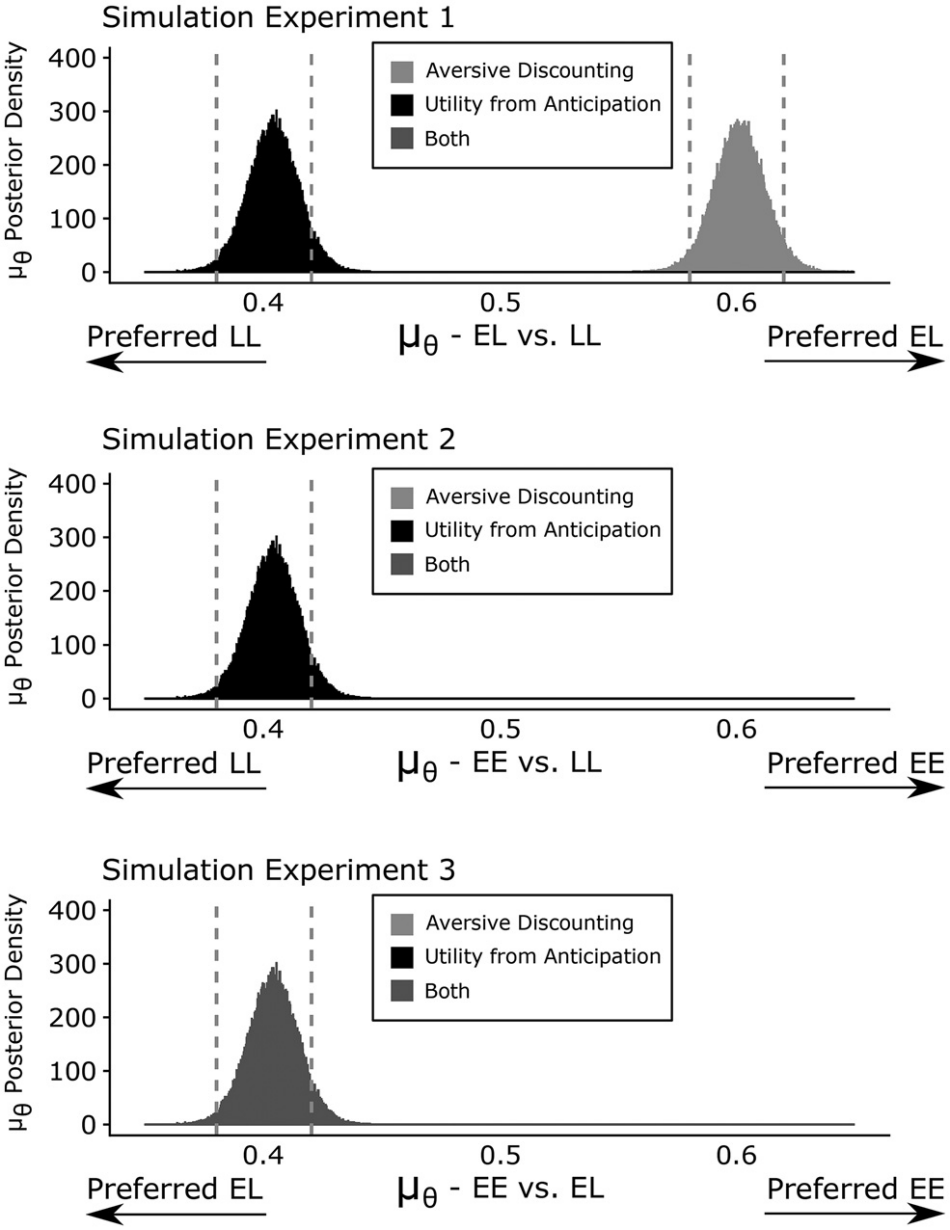
Mean parameter estimation with simulated data for all predictions according to the Bayesian hierarchy model. The predictions of both models are color coded, and on the top left corner are the specific experiments (EL: early shock + late rerwad; LL: late shock + late reward; EE: early shock + early reward). The *y*-axis shows the posterior distribution of $\mu_{\theta }$. The vertical gray lines represent the upper and lower bound for the 95% highest density interval. Experiment 1 is in the top row, and the utility from anticipation model is on the left side (upper bound = 0.42, lower bound = 0.38). On the other side is the prediction for the aversive discounting model (upper bound = 0.62, lower bound = 0.589). In the second row is experiment 2, and the prediction for the utility from anticipation model is displayed (upper bound = 0.42, lower bound = 0.38). Finally, in the bottom row, experiment 3 can be seen, and both models have the same predictions (upper bound = 0.42, lower bound = 0.38).

We used our Bayesian hierarchy model to quantitatively simulate the theory predictions (Extended Data 1; Fig. 5). Random binomial datasets were created with a probability of choosing the predicted preferred alternative of 0.6, and a probability of choosing the predicted nonpreferred alternative of 0.4. We opted for choice strengths of 0.6 or 0.4 respectively, because we considered these the weakest, yet still significant preferences above, or below, chance of one alternative over the other. As outlined in Figure 3, in experiment 1, the aversive discounting model predicts a preference for LL over EL, and the utility from anticipation model assumes a preference for EL over LL. In experiment 2, only the utility from anticipation model predicts a preference for the EE over LL. In experiment 3, both models predict a preference for EE over EL.

## Results

**Figure 6.**
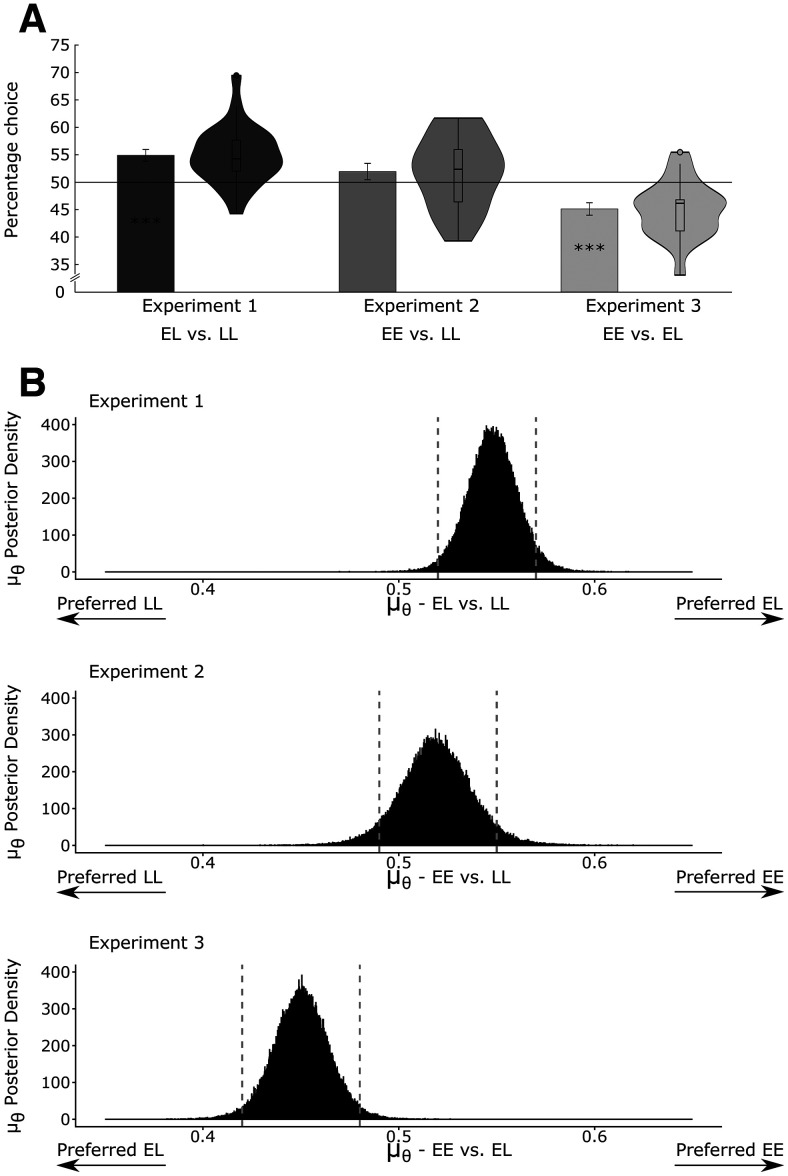
***A***, Mean percentage of decisions for each experiment. In each experiment, animals performed 10 sessions and up to 21 trials per session (6 forced trials and 16 free trials). The timings of the reward and shocks are displayed on the *x*-axis as well as the experiment. Specifically, for experiment 1, the percentage of decisions are calculated for early shock + late reward (EL) versus late shock + late reward (LL). For experiments 2 and 3, the percentage is calculated for early shock + early reward (EE) versus late shock + late reward (LL) and early shock + early reward (EE) versus early shock + early shock + late reward (EL), respectively. The vertical lines represent the SEM, and each dot represents a single animal. For all experiments, we calculated one-sample *t* tests (two-tailed). In the first experiment, animals showed a significant preference above chance level. The second experiment failed to yield any significant results; and in the third experiment, animals revealed a significant preference below chance level. The black horizontal line represents chance level. ***B***, Mean parameter estimation for the Bayesian hierarchy model. On the top left corner are the specific experiments. On the *y*-axis is the posterior distribution of $\mu_{\theta }$. Additionally, the conditions are displayed again. The vertical gray lines show the upper and lower bound for the 95% highest density interval. From top to bottom is experiment 1 (upper bound = 0.57, lower bound = 0.52), experiment 2 (upper bound = 0,55, lower bound = 0.49), and experiment 3 (upper bound = 0.48, lower bound = 0.42). See the extended data for the posterior θ distribution for all experiments (Extended Data Fig. 6-1); ***p < 0.00.

One-sample *t* tests confirmed that, in the first experiment, rats preferred the EL (mean±SEM=54.89±1.07) condition above chance level (two-tailed *t* test: *t*_(24)_ = 4.59, *p* < 0.001; Fig. 6*A*), indicating that rats preferred earlier over later shocks. Additionally, the $\mu_{\theta }$ was 0.55 with an 95% lower bound of 0.52 and an upper bound of 0.57 (Fig. 6*B*). Our diagnostics did not indicate any problems with the convergence of the Markov chain Monte Carlo (MCMC) calculations (effective chain length = 118,613, RHAT = 1, nChain = 12, Chain length = 15,000, warmup = 5000). This, tentatively, means we can trust the estimations of our simulation. The second experiment showed that rats did not significantly prefer the EE condition (mean±SEM=51.94±1.49) above chance level (two-tailed *t* test: *t*_(19)_ = 1.30, *p* = 0.210), indicating that there was no clear evidence for a preference for earlier shocks/earlier reward, or later shocks/later rewards. Bayesian analyses confirmed that rats were indeed indifferent between both alternatives. More specifically, $\mu_{\theta }$ was 0.52 with an 95% lower bound of 0.49 and an upper bound of 0.55. Hence, the point of indifference is included in the HDI. Again, the convergence of the chains (nChain = 12, Chain length = 15,000, warmup = 5000) was successful with an effective chain length of 145,906 and a RHAT = 1. Finally, in experiment 3, the EE condition (mean±SEM=45.12±1.13) was chosen significantly below chance level (two-tailed *t* test: *t*_(20)_ = 4.32, *p* < 0.001), suggesting the counterintuitive implication of a preference for the late reward over the early reward. Supporting, this implication, is the fact that $\mu_{\theta }$ was 0.45 with a 95% lower bound of 0.42 and an upper bound of 0.48. The effective chain length was 122,730 (nChain = 12, Chain length = 15,000, warmup = 5000) with a RHAT of 1.

10.1523/ENEURO.0452-21.2022.f6-1 Extended Data Figure 6-1 Posterior θ distribution for all experiments. Displayed are violin plots where the width the density represents. Additionally, boxplots are added to the violins. Download Figure 6-1, TIF file.

Thus, in summary, we found that rats chose earlier over later shocks if both rewards were equally timed. However, if entry to either arm led to an immediate shock, rats predominantly chose the arm with the late reward. This surprising finding implies that rats can be brought to choose later over sooner rewards by associating both choice alternatives with immediate shocks, thus reversing any time discounting of future reward value.

**Figure 7.**
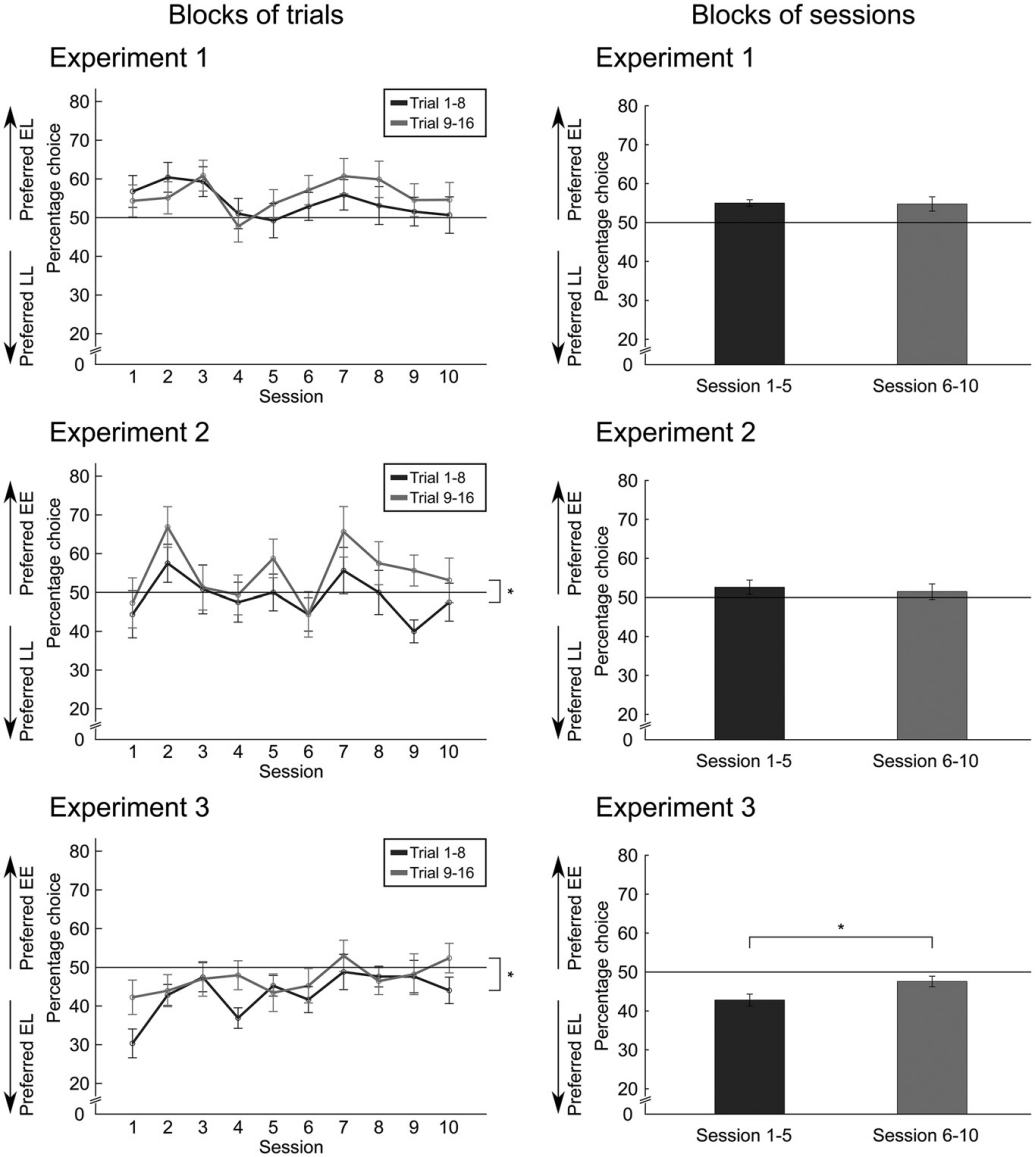
Mean percentage choice (experiment 1: early shock, late reward vs late shock, late reward, EL vs LL; experiment 2: early shock, early reward vs late shock, late reward, EE vs LL; experiment 3: early shock, early reward vs early shock, late reward, EE vs EL) for the first and second blocks of trials (trials 1–8 vs trial 9–16; left panels) and for the first and second block of sessions (sessions 1–5 vs sessions 6–10; right panels) in all experiments. Repeated-measured ANOVAs revealed significant main effects of block of trials for experiments 2 and 3. Additionally, there was a significant main effect of block of sessions on percentage choice in experiment 3, but not in experiment 1 or 2. Additionally, there were significant main effects of session order within a block on choice in experiments 1 and 2 but not in 3; *p < 0.05.

Finally, we computed additional statistics to further investigate the learning behavior (Fig. 7). We calculated the percentages of choice for the first block of trials (trials 1–8) and the second block of trials (trials 9–16). In experiment 1, the ANOVA revealed no significant block of trials × session interaction on choice (*F*_(9,207)_ = 0.740, *p *=* *0.672, *η^2^* = 0.031). The main effect of block of trials (*F*_(1,23)_ = 1.462, *p *=* *0.239, *η^2^* = 0.060) and of session (*F*_(9,207)_ = 0.950, *p *=* *0.483, *η^2^* = 0.040) on choice were not significant as well. In experiment 2, there was a no significant interaction effect of block of trials × session (*F*_(9,171)_ = 1.049, *p *=* *0.403, *η^2^* = 0.052). Additionally, no session effect was found (*F*_(9,171)_ = 1.530, *p *=* *0.141, *η^2^* = 0.075). However, there was a significant effect of block of trials (*F*_(1,19)_ = 18.832, *p *<* *0.001, *η^2^* = 0.498). Finally, in experiment 3, there was no significant block of trials × session interaction (*F*_(9,180)_ = 1.805, *p *=* *0.070, *η^2^* = 0.083). As seen before, there was no significant effect for the sessions (*F*_(4.765,95.308)_ = 1.482, *p *=* *0.205, *η^2^* = 0.069).

To check whether the behavior changed over time, we calculated the percentages of choice for the first block of sessions (sessions 1–5) and the second block of sessions (sessions 6–10). In experiment 1, there was no significant interaction of the block of sessions × session order (*F*_(4,96)_ = 0.176, *p *=* *0.950, *η^2^* = 0.007). The block of sessions showed no significant effect (*F*_(1,24)_ = 0.017, *p *=* *0.989, *η^2^* = 0.001). However, the main effect of session order within a block was significant (*F*_(4,96)_ = 2.541, *p *=* *0.045, *η^2^* = 0.096). In experiment 2, the interaction of the block of sessions × session order was not significant (*F*_(4,76)_ = 0.106, *p *= 0.980, *η^2^* = 0.006) and there was no significant effect of the block of sessions (*F*_(1,19)_ = 0.227, *p *=* *0.639, *η^2^* = 0.012). Furthermore, the main effect of session order within a block was significant (*F*_(4,76)_ = 3.883, *p *=* *0.006, *η^2^* = 0.170). The last ANOVA for experiment 3 revealed no significant interaction of the block of sessions × session order (*F*_(4,80)_ = 0.509, *p *=* *0.730, *η^2^* = 0.025). However, this time the block of sessions was significant (*F*_(1,20)_ = 7.415, *p *=* *0.013, *η^2^* = 0.270). Finally, the session order within a block had no significant influence (*F*_(4,80)_ = 1.353, *p *=* *0.258, *η^2^* = 0.063). Additionally, we compared the rats’ choice pattern in “switch” sessions, i.e., those sessions where the reward and shock contingencies changed from the previous session, with choices in “stay” sessions, i.e., sessions where the contingencies stayed the same between sessions. However, the results yielded no consistent and conclusive results across experiments. Notably, there was a higher number of switch than stay sessions across all experiments, given our randomization algorithm reported above. Hence, the inconclusiveness of this analysis might possibly because of insufficient statistical power.

## Discussion

It is well established that appetitive events are discounted over time. Thus, a reward loses value as a function of delay. However, the literature is inconsistent about the discounting of aversive events. On the one hand, the utility of anticipation model predicts an acceleration of aversive events. The model assumes that future aversive events evoke dread. On the other hand, several studies showed a discounting of aversive events similar to temporal discounting of appetitive events. Thus, aversive events should be less displeasing in the future; delayed aversive events should, hence, be preferred over earlier ones. For our three experiments, both hypotheses yield specific predictions. In the first experiment, the LL condition would be preferred according to aversive discounting and the EL condition would be preferred according to the utility from anticipation model. Our data, indeed, showed that rats significantly preferred the EL option.

Rats attach positive or negative anticipatory value to cues associated with appetitive or aversive outcomes in many contexts and situations, for instance, during place conditioning (Huston et al., 2013) or secondary reinforcement (Berridge and Robinson, 1998). In other words, associating value with stimuli predicting rewards or punishment implies that rats attribute utility to cues associated with outcomes; they thus derive utility from anticipating the outcome. It is therefore not entirely unreasonable to assume that rats derived negative utility from waiting for the shock associated with the shock-arm, and hence, interpret these data as evidence for the utility from anticipation model. However, other theories could explain this choice pattern, too: animals can make more accurate predictions of the temporal occurrence of earlier compared with later events, including shocks (Church, 2003). Hence, it has been pointed out that the preference for earlier shocks might also stem from the rats’ ability to make more accurate, and, hence, better preparatory responses for sooner than later shocks (Seligman et al., 1971).

In experiment 2, rats showed no consistent preference for EE or LL alternatives. Therefore, experiment 2 also did not provide conclusive evidence for or against either theory.

Both theories make similar predictions about the discounting of future rewards. Hence, in experiment 3, both theories, along with standard models of temporal reward discounting (Kalenscher and Pennartz, 2008) would predict choices of EE over EL options. However, contrary to these predictions, our rats preferred later over earlier rewards. This surprising finding implies that rats can be brought to choose later over sooner rewards if both choice alternatives are associated with immediate shocks, thus reversing any time discounting of future reward value.

How can we explain the somewhat surprising preference for later over sooner rewards in experiment 3? One possibility is that the temporal proximity between shock and reward matters for the evaluation of the reward: the closer in time the reward is after the shock, the less valuable it becomes. Hence, according to this idea, the shocks’ negative spill-over effects on reward values would compete with the discounting of future rewards, potentially resulting in a higher valuation of later over sooner rewards.

This hypothesis provides an alternative account of our rats’ choices in experiments 1 and 2, too. In experiment 1, rewards are farther away in time from the shocks in the EL than the LL condition. Our hypothesis would, hence, predict EL preferences, consistent with our observations. In experiment 2, there is no difference in the reward-to-shock delay between the EE and the LL options. Our hypothesis would therefore predict indifference between both alternatives, again consistent with our observations.

Interestingly, this *post hoc* hypothesis might explain some contradictory results in the literature. Knapp et al. (1959) showed that rats preferred earlier over later shocks, but Deluty (1978) found the exact opposite choice pattern. The main difference in design between those studies was the timing of reward relative to the shock; in Knapp et al. (1959), rewards were delivered at the end of a trial, after the shocks with variable delays, similar to our experiment 1. Deluty (1978), however, provided the rewards at the beginning of a trial. Thus, negative spill-over effects of shocks on the values of temporally close rewards, as hypothesized here, would result in a devaluation of the later rewards in the Knapp et al. (1959) design, but would lower the values of the sooner rewards in the Deluty (1978) design, hence explaining the differential choice patterns in both studies.

Another study (Rodríguez et al., 2018) combined a small reward with no shock and a large reward with a shock. At first, the animals preferred the small reward avoiding a shock altogether. With an increased delay between the large reward and the shock, a preference reversal occurred shifting the preference to the large reward with a shock. According to the authors, this effect can be explained with aversive discounting. However, following our hypothesis, it is also possible that the disutility of the shock lowered the value of the reward, but by increasing the delay between shock and large reward, the negative transfer effect of the shock on reward value was gradually reduced. Additionally, it was shown that the reinforcer effectiveness of aversive histamine injections decreased as a function of the delay between histamine and cocaine administrations (Woolverton et al., 2012). Interestingly, the decreased effectiveness of the aversive event was well described by a hyperboloid discounting function. Therefore, the authors argued that the results are in line with aversive discounting. However, those results are also in line with our hypothesis.

Interestingly, the data also indicate learning behavior within sessions, i.e., within each session the animals increase their preference for the chosen option. However, this pattern is not seen in experiment 1 which might be due the pseudo-randomization. Hence, the learning behavior could reflect the relearning of the side contingencies. Interestingly, we found a significant effect of session order within a block for experiment 1 and experiment 2. It is possible, that this again reflects learning behavior based on experience. However, this result has to be interpreted with caution because there was no general learning effect over all sessions. In experiment 3, the results indicate stronger preferences for the second half of the experiment. Hence, over time the animals strengthened their preference, i.e., in the first five sessions the preferences are lower compared with the last five sessions. In general, it seems like there is a complex pattern of learning within sessions and possibly over sessions as well. However, because of the pseudo-randomization and the complex pattern of significant results, those results have to interpreted with caution.

Animal experiments, such as ours, use delays in the range of several seconds. However, aversive outcomes in human intertemporal decisions are often in the range of months or years, such as negative health consequences of smoking or unhealthy life styles. It is unknown whether our results translate to longer timescales, but a recent study has shown that intertemporal decisions are comparable across different time scales (Lukinova et al., 2019). It would be intriguing to test in follow-up studies if human participants can be brought to choose delayed over early rewards when associated with differently timed punishments, albeit with much longer delays.

Finally, some authors found diverging results from ours; in analogy to our experiment 2, Renner and Specht (1967) offered rats the decision between an early shock followed by an early reward and a late shock with a late reward, among others. However, unlike in our experiment 2, their rats preferred the early shock and early reward condition. There are several procedural differences between the design by Renner and Specht (1967) and ours that may explain the differences in choice patterns. Most notably, the delay preceding the late shock, late reward in their study was much longer than our delay in experiment 2. It is possible that, with such long delays, standard reward and shock discounting mechanisms might have dominated the decision process, resulting in a devaluation of the future rewards and shocks, thus generating clear preferences. This interpretation is in line with our hypothesis since the shock-to-reward latency was kept constant across choice alternatives, and, therefore, should not matter for the choice process. In other words, it is possible that we would have found a similar choice pattern had we used longer delays, too.

In general, it is worth noting that the reasons for the discrepancy between our results and those in the literature may also be attributable to the fact that animals (Renner and Specht, 1967), much like humans (Thaler and Shefrin, 1981), discount future rewards more steeply than future aversive outcomes. Thus, depending on the magnitude and delay of the aversive and appetitive events, a rat may be biased toward deferring or accelerating the outcomes. Future studies should directly decide between the predictions of those theories.

In conclusion, we did not find unanimous support for either theory outlined in the introduction. By contrast, it seems as if decision-making between timed rewards and punishments involves at least three different mechanisms. The first two mechanisms are temporal reward and aversive discounting. As a third mechanism, we propose that shocks have a negative spill-over effect on the valuation of rewards that are close in time, with decreasing spill-over effects with increasing shock-to-reward latencies. Our theory makes the interesting and counter-intuitive prediction, supported by data from experiment 3, that rats can be brought to prefer later over sooner rewards of identical magnitudes if the later rewards are temporally decoupled from shocks. One intriguing question is if this finding could be used to nudge human participants to make more far-sighted intertemporal decisions, an implication that would have to be tested in future studies.

**Table 1:** Statistical table

Data structure	Type of test	Power
Experiment 1 Normal distribution	One-sample two-tailed *t* tests	95% confidence interval
Experiment 1 Bernoulli	Bayesian parameter estimate	95% highest density Interval
Experiment 2 Normal distribution	One-sample two-tailed *t* tests	95% confidence interval
Experiment 2 Bernoulli	Bayesian parameter estimate	95% highest density Interval
Experiment 3 Normal distribution	One-sample two-tailed *t* tests	95% confidence interval
Experiment 3 Bernoulli	Bayesian parameter estimate	95% highest density Interval

Listed are all performed tests in the same order as the text.

## Appendix

### 1. Habituation

Each animal was placed into the start box, while all doors were closed and rats received their first reward. Subsequently, all doors were opened and the animal was able to freely explore the maze. Food rewards, indicated by the reward light, were delivered after each zone transition, i.e., changing from either decision arm or the start box. After 10 min, all doors closed and the animal was removed from the maze. All animals were automatically promoted to the shaping sessions.

### Shaping

Shaping steps ensured that the rats could learn the functional principles and procedures of the task. The reward was evenly distributed between both decision arms. There was no performance criterion for any of those steps but the last.

### Step 1

The first shaping step consisted of four sessions of 16 free trials or the maximum trial duration of 30 min. In free trials, both sliding doors were opened so that rat could choose to enter either decision arm. At the beginning of the session, the animal was placed in the start box followed by food rewards. Afterwards, all doors opened and as soon as the animal entered a decision arm, all doors were closed. Fifteen seconds after entering a decision arm, food rewards were delivered. Another 10 s elapsed before the door of the chosen decision arm and the door of the start box opened. As soon as the animal entered the start arm, the door of the decision arm was closed. After entering the start box, food rewards were delivered, indicating the end of the trial.

### Step 2

The second shaping step consisted of five sessions of six forced trials and 16 free trials. The timings and structure were identical to step 1. Each session began with six forced choice trials (3 on each side in a pseudorandom order), in which the rat was directed in one of the two decision arms by just opening one of the two sliding doors. In the following 16 free choice trials, both sliding doors were opened. A session either ended after completing all trials or after 40 min.

### Step 3

Finally, the last training step was conducted, consisting of three sessions of six forced and 16 free trials. The general procedure was identical to step 2. However, 13 s after entering a decision arm, a mild electric shock (800 ms, 0.3 mA) was delivered. The shock intensity was chosen such that animals would avoid it, but it would not induce moderate or strong freezing, or any other signs of fear conditioning. The delay between shock onset and reward delivery was 1 s. The animal was removed after completion of all trials or after 40 min. Criterion for promotion to the main experiment was that each animal had passed at least 10 free trials on average over all sessions.

## Synthesis

Reviewing Editor: Michaël Zugaro, CNRS, Collège de France, Inserm Decisions are customarily a result of the Reviewing Editor and the peer reviewers coming together and discussing their recommendations until a consensus is reached. When revisions are invited, a fact-based synthesis statement explaining their decision and outlining what is needed to prepare a revision will be listed below. The following reviewer(s) agreed to reveal their identity: Jamie Roitman.

The reviewers have now agreed on a common comment on your manuscript, and are generally supportive. There are a few points that should nevertheless be addressed, as detailed below.

The present manuscript explores how delays affect the evaluation of sucessive aversive and rewarding outcomes. The authors compare two theoretical frameworks: the aversive discounting theory and the anticipation theory. One real strength of the paper lies in its simple solution to combine three experiments by varying the time to reward or the time to punishment to isolate the effects. The results are interesting (one may wonder if they are domain/species specific or more general). The paper is well written, although providing further context could help in the interpretation of the results. Overall, the authors have designed a novel task to report on an important question.

# Minor Comments

## Abstract

Well written and clear.

## Introduction

+ One aspect of utility theory is transitivity of preference. The results should be discussed in the context of this concept, particularly as the results of Experiment 3 are inconsistent with transitivity. The results would perhaps be more clearly interpretable if they were compared to conditions with reward alone (-E vs -L) and shock alone (E- vs L-) as points that would anchor the strength of the individual preferences. This relates to the issues of behavioral flexibility under conditions where there is a reversal of the contingency locations in the maze.

+ Overall the Introduction of the question draws from both learning theory of psychology and utility theory of economics. It uses some of the language of learning theory in unusual ways, e.g. aversive primary reinforcers (line 98) and aversive secondary reinforcers (line 100). Typically reinforcers - positive and negative - are stimuli that increase the likelihood of a behavior. Aversion is more typically a term referring to the hedonic value of a stimulus - for example in the realms of taste or somoatosensation. In general, the authors should exercise more care to use established terminology.

+ lines 84-92: One complication of the results that found that animals prefered sooner over later shocks is that the uncertainty of the timing of the delayed shock grows with the delay - this uncertainty may make the delayed shock more aversive, since it is closer to an “unsignaled” shock, which is well known to be more stressful than a signaled shock. This is mentioned in the discussion, but maybe merits some mention in the intro too.

+ The literature review should be expanded:

- For rats (Liley et al., 2019). https://www.ncbi.nlm.nih.gov/pmc/articles/PMC6709237/

- For humans, a huge body of literature on discounting of losses seems to be missing, such as Estle, Green, Myerson, & Holt, 2006; Mies et al., 2016; Tanaka, Yamada, Yoneda, & Ohtake, 2014. Although this body of literature covers monetary losses that are secondary reinforces, discussion of human studies that explore both secondary and primary punishments is missing (e.g., Robinson et al., 2015). https://www.frontiersin.org/articles/10.3389/fpsyg.2015.01315/full

- The authors made parallels to human decision-making, e.g.”negative health effects of smoking, or diet-related health problems “ which are on very long timescales, but their own research is on short timescale. Lukinova et al., (https://doi.org/10.7554/eLife.39656), showed that discounting decisions on the scale of seconds correlates well with decisions on the scale of days.

## Methods

+ Are the sliding doors manual or automatic?

+ line 464, 474, 481: á → of ?

+ line 148: The first sentence is confusing with respect to the abstract, which states that some experiments had different reward delays. This line seems to only apply to Experiment 1. Maybe it should start with “In Experiment 1, ...”

+ line 155-157: The logic may not be entirely clear here. How does the _utility from anticipation_ relate to the shock-to-reward delay?

+ The paper would be substantially strengthened by actually simulating the models related to the hypotheses and showing visuals, so it was clear what the predictions of the different models for different experiments would be.

+ line 208: A t-test is not ideal. A mixed effects logistic regression would be better.R-lme4

+ line 226: in the stan code `kappa = kappaMinus2 + 2`. and `kappaMinus2 ∼ gamma(0.01,0.01).

+ It would be helpful to see samples of theta_s, kappa and omega drawn from the prior to get a better sense of the model.

+ Since gamma(0.01, 0.01) is more or less 0, how can you estimate omega? it has no effect. and yet, you can. Is it possible that the data pushes kappa up just enough to estimate omega? surprising.

+ gamma in stan is specified by shape and 1/scale. Other software specifies gamma by shape and scale. It would be helpful to mention this.

+ Why does the model need the +2 and -2 in the model for transforming kappa. Did that influence convergence? initialization.

+ the author sampled a silly # of samples. 4 chains of 1000 would probably have been enough.

+ What was the warmup for the chains? (in the code, 5000)

## Results

+ The authors also refer to omission trials. It would be helpful to report the rates of omission and whether they differ across experiments.

+ The T-maze could be sketched and all experimental designs timelines supplied for better comprehension.

+ Figure 3 would be improved by adding an annotation to the x-axes of B indicating which side means what. E.g. at the left edge add “Preferred EL” and at the right edge “Preferred LL”, etc.

+ There is a typo in the y-axis of 3A “percantage” → “percetage”

+ Similar to Figure 3A dots for individual subjects (which would be better as violins), it would be nice see the posteriors of the thetas - parameters’ estimates for each animal - are distributed.

+ The # of animals, sessions and trials needs to be made clear in the text and figure caption.

+ According to one Reviewer, the main results still hold using logistic mixed effect models on the CSV files in the Bayes folder.

+ The authors focus a bit too much on the sign and significance of the effects without discussing the effect size. The rats only slightly preferred one side over the other in Exp 1 & 3.

+ There are some discrepancies between some of the values in the excel files and those in the CSV. On line 39 of ExperimentalBook_Experiment_3_Batch_1.xlsx that is meant to be data from Experiment 3, Animal 2, Session 3. In the excel sheet there were 5 EE and 11 EL, but the CSV had different data. After more digging, it appears that the Animal numbers in the excel files didn’t match - because they were incremented depending on the batch #. Figuring this out caused quite a waste of time. The authors might have at least mentioned that in the ReadMe.txt.

+ The Bayesplot R package is a good recommendation for nice plots of stan models.

In addition, the experimental design is not completely clear. It appears that each of the 3 experiments is conducted in a separate group of rats. How many sessions does each animal perform? Do they perform 1 session (22 trials) per day? The structure of the experimental sessions (6 forced trials followed by 16 free) raises the possibility that choices may be affected by the immediate prior outcome contingencies. How is stability of behavior assessed to be sure that behavioral flexibility is not affecting the results?

In order to strengthen the paper and provide a more comprehensive description of the data, the authors may wish to perform additional analyses to evaluate a potential contribution of learning/adaptation effects on choices. The authors have trials 1-8 (first half) and 9-16 (second half) for sessions 1-10 (less to more experience). They could further mark each session as maintaining or reversing the arm assignments from the prior session. The authors could make a number of comparisons that could potentially quantify the contributions of learning/adaptation to choice patterns, e.g.

- first vs. second half across all sessions

- first half in a switch session vs. second half in maintained

- second half with less (sessions 1-4) vs. more (sessions 7-10) experience

Currently the results are aggregated which may lose the potential dynamics of behavior that might exist within the data set that arise from ongoing experience with the task. Instead, it would be interesting to quantify the effects not only of the timing of rewards/shocks, but also session half, session number, and whether the session requires a reversal of behavior from the prior session. This would not be expected to alter the main message of the paper, but would provide a more comprehensive description of the data.

## Discussion

Interesting discussion.

## Author Response

Synthesis Statement for Author (Required):

The reviewers have now agreed on a common comment on your manuscript, and are generally supportive. There are a few points that should nevertheless be addressed, as detailed below.

The present manuscript explores how delays affect the evaluation of sucessive aversive and rewarding outcomes. The authors compare two theoretical frameworks: the aversive discounting theory and the anticipation theory. One real strength of the paper lies in its simple solution to combine three experiments by varying the time to reward or the time to punishment to isolate the effects. The results are interesting (one may wonder if they are domain/species specific or more general). The paper is well written, although providing further context could help in the interpretation of the results. Overall, the authors have designed a novel task to report on an important question.

Our reply: We thank the editor and the reviewers for their generally favourable assessment of our submission. We have addressed all critical comments below.

# Minor Comments

## Abstract

Well written and clear.

## Introduction

+ One aspect of utility theory is transitivity of preference. The results should be discussed in the context of this concept, particularly as the results of Experiment 3 are inconsistent with transitivity. The results would perhaps be more clearly interpretable if they were compared to conditions with reward alone (-E vs -L) and shock alone (E- vs L-) as points that would anchor the strength of the individual preferences. This relates to the issues of behavioral flexibility under conditions where there is a reversal of the contingency locations in the maze.

Our reply: we thank the reviewer for their comment. However, with respect, we would like to point out that our rats’ choices did satisfy transitivity, at least at face value (cf. fig. 6): they chose EL over LL (exp. 1), were indifferent between LL and EE (exp. 2), and chose EL over EE (exp. 3). Thus, EL was indirectly revealed preferred over EE across the choice chain. We realize that this misunderstanding might have come up because we may not have presented our rats’ choice pattern in the most accessible way. We apologize for this and have now changed fig. 6 to make it easier for the reader to see the rats’ choices. In addition, however, please also note that our experiment was a between-subject design, i.e., we used different animals for each experiment. In order to check for strong or weak transitivity, it would be necessary to test individual rats in a within-subject design across all conditions. This is an exciting idea that we consider testing in follow-up studies.

The reviewer also suggested to compare the rats’ choices in conditions with only reward, or only shock. For only-rewards, we would expect clear preferences for earlier over later rewards, as seen in standard delay discounting tasks (note that the reward magnitude was always identical, they only differed in delay). However, we are not sure that the only-shock condition could work: it would be difficult to motivate rats to choose between early and late shocks if they were not associated with reward; we would expect rats to stop performing the task, and/or to freeze. We therefore did not run the suggested experiments. However, we would be happy to add those experiments should the reviewer continue to feel that they are necessary.

+ Overall the Introduction of the question draws from both learning theory of psychology and utility theory of economics. It uses some of the language of learning theory in unusual ways, e.g. aversive primary reinforcers (line 108) and aversive secondary reinforcers (line 110). Typically reinforcers - positive and negative - are stimuli that increase the likelihood of a behavior. Aversion is more typically a term referring to the hedonic value of a stimulus - for example in the realms of taste or somoatosensation. In general, the authors should exercise more care to use established terminology.

Our reply: We thank the reviewer and agree with their comment. We changed the terminology to be more consistent with the established language in learning theory and economics.

+ lines 84-92: One complication of the results that found that animals prefered sooner over later shocks is that the uncertainty of the timing of the delayed shock grows with the delay - this uncertainty may make the delayed shock more aversive, since it is closer to an “unsignaled” shock, which is well known to be more stressful than a signaled shock. This is mentioned in the discussion, but maybe merits some mention in the intro too.

Our reply: We fully agree with the reviewer. We have therefore added the following section to the introduction (line 99-102):

“However, note that an alternative interpretation of the rats’ preference for early over late shocks is that the uncertainty of the timing of the shock increases with increasing delay. Animals may prefer sooner over later shocks to make more appropriate preparatory responses (Knapp et al., 1959; Seligman et al., 1971).”

+ The literature review should be expanded:

- For rats (Liley et al., 2019). https://www.ncbi.nlm.nih.gov/pmc/articles/PMC6709237/

- For humans, a huge body of literature on discounting of losses seems to be missing, such as Estle, Green, Myerson, & Holt, 2006; Mies et al., 2016; Tanaka, Yamada, Yoneda, & Ohtake, 2014. Although this body of literature covers monetary losses that are secondary reinforces, discussion of human studies that explore both secondary and primary punishments is missing (e.g., Robinson et al., 2015). https://www.frontiersin.org/articles/10.3389/fpsyg.2015.01315/full

- The authors made parallels to human decision-making, e.g.”negative health effects of smoking, or diet-related health problems “ which are on very long timescales, but their own research is on short timescale. Lukinova et al., (https://doi.org/10.7554/eLife.39656), showed that discounting decisions on the scale of seconds correlates well with decisions on the scale of days.

Our reply: We have added those references, as well as text sections covering those references, to several parts in our manuscript:

Introduction, line 60-61: “This theory was empirically supported by several studies in rats who preferred later over earlier electric shocks (Deluty, 1978; Liley et al., 2019).”

Introduction, line 85-93: “In another study, human participants preferred a smaller, sooner over a larger, later monetary loss (Holt et al., 2008; Thaler and Shefrin, 1981). In general, humans discount delayed gains more steeply than delayed losses indicating different processes for the discounting of positive and negative outcomes (Estle et al., 2006; Mies et al., 2016). This effect is called the sign effect and participants exhibiting the sign effect show different neural activity compared to participants not experiencing the sign effect (Tanaka et al., 2014). Sign effects are observed for monetary losses, but also primary punishers like the threat of a shock (Berns et al., 2006; Robinson et al., 2015).”

Discussion, line 470-477: “Animal experiments, such as ours, use delays in the range of several seconds. However, aversive outcomes in human intertemporal decisions are often in the range of months or years, such as negative health consequences of smoking or unhealthy life styles. It is unknown if our results translate to longer timescales, but a recent study has shown that intertemporal decisions are comparable across different time scales (Lukinova et al., 2019). It would be intriguing to test in follow-up studies if human participants can be brought to choose delayed over early rewards when associated with differently timed punishments, albeit with much longer delays.”

## Methods

+ Are the sliding doors manual or automatic?

Our reply: the doors were automatic. We have now added this information to the manuscript (line 140).

+ line 464, 474, 481: á → of ?

Our reply: we have replaced à with of throughout the entire manuscript and in the appendix.

+ line 148: The first sentence is confusing with respect to the abstract, which states that some experiments had different reward delays. This line seems to only apply to Experiment 1. Maybe it should start with “In Experiment 1, ...”

Our reply: We have made the requested change (line 165-168).

+ line 155-15

Finally, the last training step was conducted, consisting of three sessions of six forced and 16 free trials. The general procedure was identical to step 2. However, 13 s after entering a decision arm, a mild electric shock (800 ms, 0.3 mA) was delivered. The shock intensity was chosen such that animals would avoid it, but it would not induce moderate or strong freezing, or any other signs of fear conditioning. The delay between shock onset and reward delivery was 1 s. The animal was removed after completion of all trials or after 40 min. Criterion for promotion to the main experiment was that each animal had passed at least 10 free trials on average over all sessions.

7: The logic may not be entirely clear here. How does the _utility from anticipation_ relate to the shock-to-reward delay?

Our reply: we have expanded the relevant section and explain in greater detail the logic of the different theory predictions as well as the potential implications of the confound in our design with respect to those predictions, and how we addressed this confound. The section now reads (line 171-190):

“The aversive discounting theory predicts that rats would choose the arm yielding a late shock because the negative value of late shocks should be discounted; late shocks should, thus, be less aversive than early shocks at the time point of decision. By contrast, the utility from anticipation theory would predict choices of the early shock because of increasing dread with longer delays.

Note that this experimental design confounds entry-to-shock delay (the delay between entering the arm and receiving the shock) with shock-to-reward delay (late shocks are closer in time to reward than early shocks). A choice of late rewards could be indicative of utility from anticipation, as outlined above, but, given this confound, it is equally plausible that rats might use the shock as a cue to predict the following reward; hence, if this was true, the delay between cue and reward would be shorter in LL trials, temporal reward discounting would therefore predict choices of the late shock.”

+ The paper would be substantially strengthened by actually simulating the models related to the hypotheses and showing visuals, so it was clear what the predictions of the different models for different experiments would be.

Our reply: we ran the requested simulations and a new section and a new figure; see chapter 2.8 and figure 5, lines 279-288:

“We used our Bayesian hierarchy model to quantitatively simulate the theory predictions (figure 5). Random binomial data sets were created with a probability of choosing the predicted preferred alternative of 0.6, and a probability of choosing the predicted non-preferred alternative of 0.4. We opted for choice strengths of 0.6, or 0.4 respectively, because we considered these the weakest, yet still significant preferences above, or below, chance of one alternative over the other. As outlined in figure 3, in experiment 1, the aversive discounting model predicts a preference for LL over EL, and the utility from anticipation model assumes a preference for EL over LL. In experiment 2, only the utility from anticipation model predicts a preference for the EE over LL. In Experiment 3, both models predict a preference for EE over EL.

Figure 5. Mean parameter estimation with simulated data for all predictions according to the Bayesian hierarchy model. The predictions of both models are color coded and on the top left corner are the specific experiments. The y-axis shows the posterior distribution of μ_θ. The vertical gray lines represent the upper and lower bound for the 95% highest density interval. Experiment 1 is in the top row and the utility from anticipation model is on the left side (upper bound = 0.42, lower bound = 0.38). On the other side is the prediction for the aversive discounting model (upper bound = 0.62, lower bound = 0.589). In the second row is experiment 2 and the prediction for the utility from anticipation model is displayed (upper bound = 0.42, lower bound = 0.38). Finally, in the bottom row experiment 3 can be seen and both models have the same predictions (upper bound = 0.42, lower bound = 0.38).

+ line 208: A t-test is not ideal. A mixed effects logistic regression would be better.R-lme4

Our reply: With respect, we are not sure we fully understand the reviewer’s suggestion. Since we used a between-subject design, we would have to run three separate logistic regressions for each experiment, with condition (e.g., EL vs. LL in experiment 1) as categorical predictor, and choice (left vs. right) as dependent variable. However, such logistic regressions would yield nearly the same results as our t-tests. We will be very happy to run a mixed logistic regression, but would greatly appreciate if the reviewer could provide some additional details on their suggestion.

+ line 226: in the stan code `kappa = kappaMinus2 + 2`. and `kappaMinus2 ∼ gamma(0.01,0.01).

Our reply: This Transformation is needed because the beta function of θ was μ_θ (κ-2)+1,(1- μ_θ)(κ-2)+1 and κ needs to be non-negative. Hence, `kappaMinus2` is modeled by gamma(0.01,0.01) and kappa = kappaMinus2 +2. Therefore, (κ-2) of the beta function is always non-negative. (See Kruschke, J. (2014). Doing Bayesian data analysis: A tutorial with R, JAGS, and Stan; page 127-132 and pages 235-236)

https://nyu-cdsc.github.io/learningr/assets/kruschke_bayesian_in_R.pdf

+ It would be helpful to see samples of theta_s, kappa and omega drawn from the prior to get a better sense of the model.

Our reply: The prior distributions are illustrated in Figure 2. Additionally, we drew actually priors and added those graphs to the extended data (Figure 4-1 - 4-3).

+ Since gamma(0.01, 0.01) is more or less 0, how can you estimate omega? it has no effect. and yet, you can. Is it possible that the data pushes kappa up just enough to estimate omega? surprising.

Our reply: This gamma distribution is strongly left skewed and we therefore trust that the gamma distribution yields sufficiently strong predictions for low values. Because gamma(0.01,0.01) is quite low, the estimation of omega (based on the beta function) is really vague. In order to be able to see the prior values of the kappa distribution, a histogram was added to the extended data (figure 4-1). In general, with stronger prior belief about omega, we would choose a larger kappa (different gamma distribution).

+ gamma in stan is specified by shape and 1/scale. Other software specifies gamma by shape and scale. It would be helpful to mention this.

Our reply: A comment was added to the stan file.

+ Why does the model need the +2 and -2 in the model for transforming kappa. Did that influence convergence? initialization.

Our reply: We opted for these transformation to ensure that kappa is non-negative. Please see our reply to the previous comment above for more details.

+ the author sampled a silly # of samples. 4 chains of 1000 would probably have been enough.

Our reply: Thank you for your comment. We made sure to conform to the recommendations on chain lengths specified in Kruschke, J. (2014; Doing Bayesian data analysis: A tutorial with R, JAGS, and Stan); ESS is recommended to be around ∼10000. Additionally, the number of chains was based on the number of cores of the used CPU (Real cores; Not threads). Since only the model was running, there was no reason to spare any CPU resources. However, for replications of our results, we may consider using shorter chain length. For Experiment 1 and 3, the suggested chains and length worked as well as shorter lengths, and the results were the same (Warmup = 200 steps). However, for some reason this was not true for Experiment 2. Without sufficient warmup, it seems like (at least) one chain gets stuck and it appears that at least a warmup of 2000 steps and 1000 regular steps.

+ What was the warmup for the chains? (in the code, 5000)

Our reply: Yes, the warmup length was 5000, we apologize for not mentioning this. We have added this information to the manuscript.

## Results

+ The authors also refer to omission trials. It would be helpful to report the rates of omission and whether they differ across experiments.

Our reply: the omission trials were consistently below 1%. We have added the information in Line 242-246:

“In all three experiments, animals performed nearly all trials (Experiment 1: 99,20%; Experiment 2: 99.97%; Experiment 3: 99.85%). Additionally, trials in which animals avoided the shock were labelled as omission trials. However, because of the low occurrence rate (Experiment 1: 0.80%; Experiment 2: 0.02%; Experiment 3: 0.13%), we excluded them from analysis. “

+ The T-maze could be sketched and all experimental designs timelines supplied for better comprehension.

Our reply: A sketch of the T-Maze was added to the manuscript as well as an overview of the timings for shocks and rewards (see chapter 2.2 and 2.3).

+ Figure 3 would be improved by adding an annotation to the x-axes of B indicating which side means what. E.g. at the left edge add “Preferred EL” and at the right edge “Preferred LL”, etc.

Our reply: We agree and now have added annotations to the figure.

+ There is a typo in the y-axis of 3A “percantage” → “percetage”

Our reply: we corrected the typo.

+ Similar to Figure 3A dots for individual subjects (which would be better as violins), it would be nice see the posteriors of the thetas - parameters’ estimates for each animal - are distributed.

Our reply: The posteriors of the theta parameter are now added as a violin plot to the extended data (Figure 6-1). Additionally, figure 6A (former: figure 3A) in the main MS was changed to violin plots for consistency.

+ The # of animals, sessions and trials needs to be made clear in the text and figure caption.

Our reply: We have added this information to the text and the figure captions, where relevant. In addition, we have added information about the performed trials (Line 242-246).

+ According to one Reviewer, the main results still hold using logistic mixed effect models on the CSV files in the Bayes folder.

Our reply: we would be very happy to run those analyses, and include them in our MS, but we need some more information from the reviewer. We would therefore appreciate if they could provide those information (which predictors/factors etc.), or even provide the scripts they used.

+ The authors focus a bit too much on the sign and significance of the effects without discussing the effect size. The rats only slightly preferred one side over the other in Exp 1 & 3.

Our reply: We acknowledge that our interpretation and conclusion of our data focuses mainly on the sign and significance of the rats’ choices, even though the preference strength for one choice alternative over the other was quite small in all experiments. We believe that there are several possible reasons for the absence of strong preferences. First, the association between the arms in the T-maze and the outcomes (shock and reward delays) was pseudo-randomized across rats and days, so that rats had to relearn the outcome contingencies anew almost every day, driving variability in choice. Second, rats rarely express strong preferences for one alternative over the other because of their innate tendency to balance exploration and exploitation, resulting in often alternating choice behavior. However, we believe that this is not a big problem. Our Bayesian analyses quantify the level of evidence for or against our hypotheses, so they should be robust against this variability in choice.

+ There are some discrepancies between some of the values in the excel files and those in the CSV. On line 39 of ExperimentalBook_Experiment_3_Batch_1.xlsx that is meant to be data from Experiment 3, Animal 2, Session 3. In the excel sheet there were 5 EE and 11 EL, but the CSV had different data. After more digging, it appears that the Animal numbers in the excel files didn’t match - because they were incremented depending on the batch #. Figuring this out caused quite a waste of time. The authors might have at least mentioned that in the ReadMe.txt.

Our reply: we sincerely apologize for this mishap, we simply forgot to mention it. We added a note in the ReadMe.txt so this won’t happen again.

+ The Bayesplot R package is a good recommendation for nice plots of stan

Our reply: Thank you for the tip!

In addition, the experimental design is not completely clear. It appears that each of the 3 experiments is conducted in a separate group of rats. How many sessions does each animal perform? Do they perform 1 session (22 trials) per day? The structure of the experimental sessions (6 forced trials followed by 16 free) raises the possibility that choices may be affected by the immediate prior outcome contingencies.

Our reply: we have added the information on sessions and trials to our methods section 2.5 (lines 218-220).

How is stability of behavior assessed to be sure that behavioral flexibility is not affecting the results? In order to strengthen the paper and provide a more comprehensive description of the data, the authors may wish to perform additional analyses to evaluate a potential contribution of learning/adaptation effects on choices. The authors have trials 1-8 (first half) and 9-16 (second half) for sessions 1-10 (less to more experience). They could further mark each session as maintaining or reversing the arm assignments from the prior session. The authors could make a number of comparisons that could potentially quantify the contributions of learning/adaptation to choice patterns, e.g.

- first vs. second half across all sessions

- first half in a switch session vs. second half in maintained

- second half with less (sessions 1-4) vs. more (sessions 7-10) experience

Currently the results are aggregated which may lose the potential dynamics of behavior that might exist within the data set that arise from ongoing experience with the task. Instead, it would be interesting to quantify the effects not only of the timing of rewards/shocks, but also session half, session number, and whether the session requires a reversal of behavior from the prior session. This would not be expected to alter the main message of the paper, but would provide a more comprehensive description of the data.

Line 248-254: To test for learning effects, for each experiment, we ran a repeated-measures ANOVA of the effect of trial block (block 1: trials 1-8 vs. block 2: trials 9-16) and session number on the percentage of choice. A second repeated-measures ANOVA (within-subject-factors: first vs. second block of sessions, i.e., block 1: sessions 1-5 vs. block 2: sessions 6-10; session order within each block) was calculated to check whether the behavior changed over time.

Line 348-376: Finally, we computed additional statistics to further investigate the learning behavior (Figure 7). We calculated the percentages of choice for the first half of trials (Trial 1-8) and the second half of trials (Trial 9-16). In experiment 1, the ANOVA revealed no significant block of trials*session interaction on choice (F_([9,207]) = 0.740, p = 0.672, η² = 0.031). The main effect of block of trials (F_([1,23]) = 1.462, p = 0.239, η² = 0.060) and of session (F_([9,207]) = 0.950, p = 0.483, η² = 0.040) on choice were not significant as well. In Experiment 2, there was a no significant interaction effect of block of trials *session (F_([9,171]) = 1.049, p = 0.403, η² = 0.052). Additionally, no session effect was found (F_([9,171]) = 1.530, p = 0.141, η² = 0.075). However, there was a significant effect of block of trials (F_([1,19]) = 18.832, p < 0.001, η² = 0.498). Lastly, in experiment 3, there was no significant block of trials *session interaction (F_([9,180]) = 1.805, p = 0.070, η² = 0.083). As seen before, there was no significant effect for the sessions (F_([4.765,95.308]) = 1.482, p = 0.205, η² = 0.069). Again, the main effect of the block of trials was significant (F_([1,20]) = 6.369, p = 0.020, η² = 0.242).

Line 456-469: Interestingly, the data also indicates learning behavior within sessions, i.e. within each session the animals increase their preference for the chosen option. However, this pattern is not seen in Experiment 1 which might be due the pseudo-randomization. Hence, the learning behavior could reflect the relearning of the side contingencies. Interestingly, we found a significant effect of session order within a block for experiment 1 and experiment 2. It is possible, that this again reflects learning behavior based on experience. However, this result has to be interpreted with caution because there was no general learning effect over all sessions. In experiment 3, the results indicate stronger preferences for the second half of the experiment. Hence, over time the animals strengthened their preference, i.e. in the first five sessions the preferences are lower compared to the last five sessions. In general, it seems like there is a complex pattern of learning within sessions and possibly over sessions as well. However, because of the pseudo-randomization and the complex pattern of significant results, those results have to interpreted with caution.

## Discussion

Interesting discussion.
