# Supplementary material for: Discounting of Future Rewards and Punishments in Rats
Source: eNeuro. 2022 Nov 30;9(6):ENEURO.0452-21.2022. doi: 10.1523/ENEURO.0452-21.2022 (PMC9718352; doi:10.1523/ENEURO.0452-21.2022)

General Linear Model

Notes

Output Created		13-JUL-2022 02:22:33
Comments		
Input	Data	H:\sciebo\AA Discounting\Paper_Behavior\eNeuro\Revision_2_0\Data_and_Codes\NHST\AA_Disc_Behavior_Data_v_1_3.sav
	Active Dataset	DataSet1
	Filter	<none>
	Weight	<none>
	Split File	<none>
	N of Rows in Working Data File	25
Missing Value Handling	Definition of Missing	User-defined missing values are treated as missing.
	Cases Used	Statistics are based on all cases with valid data for all variables in the model.
Syntax		GLM Experiment_1A_Switch_Mean_Block_1 Experiment_1A_Switch_Mean_Block_2<br><br>Experiment_1A_Stay_Mean_Block_1 Experiment_1A_Stay_Mean_Block_2<br>/WSFACTOR=Stay 2 Polynomial Block 2 Polynomial<br>/METHOD=SSTYPE(3)<br>/PLOT=PROFILE (Block*Stay) TYPE=BAR ERRORBAR=SE(1) MEANREFERENCE=NO<br>/PRINT=DESCRIPTIVE ETASQ<br>/CRITERIA=ALPHA(.05)<br>/WSDESIGN=Stay Block Stay*Block.
Resources	Processor Time	00:00:00,16
	Elapsed Time	00:00:00,10

Within-Subjects Factors

Measure: MEASURE_1

Stay	Block	Dependent Variable
1	1	Experiment_1A_Switch_Mean_Block_1
	2	Experiment_1A_Switch_Mean_Block_2
2	1	Experiment_1A_Stay_Mean_Block_1
	2	Experiment_1A_Stay_Mean_Block_2

Descriptive Statistics

	Mean	Std. Deviation	N
Experiment_1A_Switch_Mean_Block_1	54.6796	9.47835	25
Experiment_1A_Switch_Mean_Block_2	55.2482	7.81221	25
Experiment_1A_Stay_Mean_Block_1	50.8750	11.17520	25
Experiment_1A_Stay_Mean_Block_2	56.8294	10.81637	25

Multivariate Tests[a]

Effect		Value	F	Hypothesis df	Error df	Sig.
Stay	Pillai's Trace	.009	.206[b]	1.000	24.000	.654
	Wilks' Lambda	.991	.206[b]	1.000	24.000	.654
	Hotelling's Trace	.009	.206[b]	1.000	24.000	.654
	Roy's Largest Root	.009	.206[b]	1.000	24.000	.654
Block	Pillai's Trace	.156	4.436[b]	1.000	24.000	.046
	Wilks' Lambda	.844	4.436[b]	1.000	24.000	.046
	Hotelling's Trace	.185	4.436[b]	1.000	24.000	.046
	Roy's Largest Root	.185	4.436[b]	1.000	24.000	.046
Stay * Block	Pillai's Trace	.134	3.698[b]	1.000	24.000	.066
	Wilks' Lambda	.866	3.698[b]	1.000	24.000	.066
	Hotelling's Trace	.154	3.698[b]	1.000	24.000	.066
	Roy's Largest Root	.154	3.698[b]	1.000	24.000	.066

Multivariate Tests[a]

Effect		Partial Eta Squared
Stay	Pillai's Trace	.009
	Wilks' Lambda	.009
	Hotelling's Trace	.009
	Roy's Largest Root	.009
Block	Pillai's Trace	.156
	Wilks' Lambda	.156
	Hotelling's Trace	.156
	Roy's Largest Root	.156
Stay * Block	Pillai's Trace	.134
	Wilks' Lambda	.134
	Hotelling's Trace	.134
	Roy's Largest Root	.134

a. Design: Intercept
Within Subjects Design: Stay + Block + Stay * Block

b. Exact statistic

Mauchly's Test of Sphericity[a]

Measure: MEASURE_1

Within Subjects Effect	Mauchly's W	Approx. Chi-Square	df	Sig.	Epsilon[b] Greenhouse-Geisser
Stay	1.000	.000	0	.	1.000
Block	1.000	.000	0	.	1.000
Stay * Block	1.000	.000	0	.	1.000

Mauchly's Test of Sphericity[a]

Measure: MEASURE_1

	Epsilon[b]	
Within Subjects Effect	Huynh-Feldt	Lower-bound
Stay	1.000	1.000
Block	1.000	1.000
Stay * Block	1.000	1.000

Tests the null hypothesis that the error covariance matrix of the orthonormalized transformed dependent variables is proportional to an identity matrix.

a. Design: Intercept
Within Subjects Design: Stay + Block + Stay * Block

b. May be used to adjust the degrees of freedom for the averaged tests of significance. Corrected tests are displayed in the Tests of Within-Subjects Effects table.

Tests of Within-Subjects Effects

Measure: MEASURE_1

Source		Type III Sum of Squares	df	Mean Square	F
Stay	Sphericity Assumed	30.898	1	30.898	.206
	Greenhouse-Geisser	30.898	1.000	30.898	.206
	Huynh-Feldt	30.898	1.000	30.898	.206
	Lower-bound	30.898	1.000	30.898	.206
Error(Stay)	Sphericity Assumed	3600.395	24	150.016	
	Greenhouse-Geisser	3600.395	24.000	150.016	
	Huynh-Feldt	3600.395	24.000	150.016	
	Lower-bound	3600.395	24.000	150.016	
Block	Sphericity Assumed	265.934	1	265.934	4.436
	Greenhouse-Geisser	265.934	1.000	265.934	4.436
	Huynh-Feldt	265.934	1.000	265.934	4.436
	Lower-bound	265.934	1.000	265.934	4.436
Error(Block)	Sphericity Assumed	1438.913	24	59.955	
	Greenhouse-Geisser	1438.913	24.000	59.955	
	Huynh-Feldt	1438.913	24.000	59.955	
	Lower-bound	1438.913	24.000	59.955	
Stay * Block	Sphericity Assumed	181.289	1	181.289	3.698
	Greenhouse-Geisser	181.289	1.000	181.289	3.698
	Huynh-Feldt	181.289	1.000	181.289	3.698
	Lower-bound	181.289	1.000	181.289	3.698
Error(Stay*Block)	Sphericity Assumed	1176.571	24	49.024	
	Greenhouse-Geisser	1176.571	24.000	49.024	
	Huynh-Feldt	1176.571	24.000	49.024	
	Lower-bound	1176.571	24.000	49.024	

Tests of Within-Subjects Effects

Measure: MEASURE_1

Source		Sig.	Partial Eta Squared
Stay	Sphericity Assumed	.654	.009
	Greenhouse-Geisser	.654	.009
	Huynh-Feldt	.654	.009
	Lower-bound	.654	.009
Error(Stay)	Sphericity Assumed		
	Greenhouse-Geisser		
	Huynh-Feldt		
	Lower-bound		
Block	Sphericity Assumed	.046	.156
	Greenhouse-Geisser	.046	.156
	Huynh-Feldt	.046	.156
	Lower-bound	.046	.156
Error(Block)	Sphericity Assumed		
	Greenhouse-Geisser		
	Huynh-Feldt		
	Lower-bound		
Stay * Block	Sphericity Assumed	.066	.134
	Greenhouse-Geisser	.066	.134
	Huynh-Feldt	.066	.134
	Lower-bound	.066	.134
Error(Stay*Block)	Sphericity Assumed		
	Greenhouse-Geisser		
	Huynh-Feldt		
	Lower-bound		

Tests of Within-Subjects Contrasts

Measure: MEASURE_1

Source	Stay	Block	Type III Sum of Squares	df	Mean Square	F
Stay	Linear		30.898	1	30.898	.206
Error(Stay)	Linear		3600.395	24	150.016	
Block		Linear	265.934	1	265.934	4.436
Error(Block)		Linear	1438.913	24	59.955	
Stay * Block	Linear	Linear	181.289	1	181.289	3.698
Error(Stay*Block)	Linear	Linear	1176.571	24	49.024	

Tests of Within-Subjects Contrasts

Measure: MEASURE_1

Source	Stay	Block	Sig.	Partial Eta Squared
Stay	Linear		.654	.009
Error(Stay)	Linear			
Block		Linear	.046	.156
Error(Block)		Linear		
Stay * Block	Linear	Linear	.066	.134
Error(Stay*Block)	Linear	Linear		

Tests of Between-Subjects Effects

Measure: MEASURE_1

Transformed Variable: Average

Source	Type III Sum of Squares	df	Mean Square	F	Sig.	Partial Eta Squared
Intercept	296023.512	1	296023.512	2213.198	<,001	.989
Error	3210.090	24	133.754			

Profile Plots

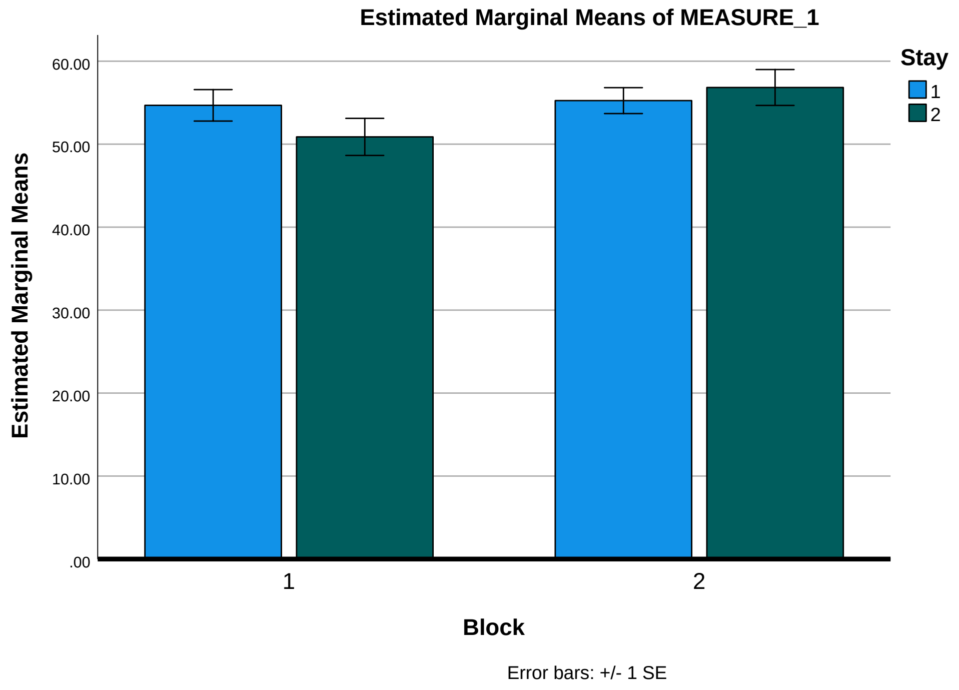

General Linear Model

Notes

Output Created		13-JUL-2022 02:22:33
Comments		
Input	Data	H:\sciebo\AA Discounting\Paper_Behavior\eNeuro\Revision_2_0\Data_and_Codes\NHST\AA_Disc_Behavior_Data_v_1_3.sav
	Active Dataset	DataSet1
	Filter	<none>
	Weight	<none>
	Split File	<none>
	N of Rows in Working Data File	25
Missing Value Handling	Definition of Missing	User-defined missing values are treated as missing.
	Cases Used	Statistics are based on all cases with valid data for all variables in the model.
Syntax		GLM Experiment_1B_Switch_Mean_Block_1 Experiment_1B_Switch_Mean_Block_2<br><br>Experiment_1B_Stay_Mean_Block_1 Experiment_1B_Stay_Mean_Block_2<br>/WSFACTOR=Stay 2 Polynomial Block 2 Polynomial<br>/METHOD=SSTYPE(3)<br>/PLOT=PROFILE (Block*Stay) TYPE=BAR ERRORBAR=SE(1) MEANREFERENCE=NO<br>/PRINT=DESCRIPTIVE ETASQ<br>/CRITERIA=ALPHA(.05)<br>/WSDESIGN=Stay Block Stay*Block.
Resources	Processor Time	00:00:00,13
	Elapsed Time	00:00:00,10

Within-Subjects Factors

Measure: MEASURE_1

Stay	Block	Dependent Variable
1	1	Experiment_1B_Switch_Mean_Block_1
	2	Experiment_1B_Switch_Mean_Block_2
2	1	Experiment_1B_Stay_Mean_Block_1
	2	Experiment_1B_Stay_Mean_Block_2

Descriptive Statistics

	Mean	Std. Deviation	N
Experiment_1B_Switch_Mean_Block_1	46.3371	7.29418	20
Experiment_1B_Switch_Mean_Block_2	53.4650	10.20809	20
Experiment_1B_Stay_Mean_Block_1	54.5238	12.44593	20
Experiment_1B_Stay_Mean_Block_2	61.0938	13.98472	20

Multivariate Tests[a]

Effect		Value	F	Hypothesis df	Error df	Sig.
Stay	Pillai's Trace	.302	8.201[b]	1.000	19.000	.010
	Wilks' Lambda	.698	8.201[b]	1.000	19.000	.010
	Hotelling's Trace	.432	8.201[b]	1.000	19.000	.010
	Roy's Largest Root	.432	8.201[b]	1.000	19.000	.010
Block	Pillai's Trace	.486	17.935[b]	1.000	19.000	<,001
	Wilks' Lambda	.514	17.935[b]	1.000	19.000	<,001
	Hotelling's Trace	.944	17.935[b]	1.000	19.000	<,001
	Roy's Largest Root	.944	17.935[b]	1.000	19.000	<,001
Stay * Block	Pillai's Trace	.001	.016[b]	1.000	19.000	.900
	Wilks' Lambda	.999	.016[b]	1.000	19.000	.900
	Hotelling's Trace	.001	.016[b]	1.000	19.000	.900
	Roy's Largest Root	.001	.016[b]	1.000	19.000	.900

Multivariate Tests[a]

Effect		Partial Eta Squared
Stay	Pillai's Trace	.302
	Wilks' Lambda	.302
	Hotelling's Trace	.302
	Roy's Largest Root	.302
Block	Pillai's Trace	.486
	Wilks' Lambda	.486
	Hotelling's Trace	.486
	Roy's Largest Root	.486
Stay * Block	Pillai's Trace	.001
	Wilks' Lambda	.001
	Hotelling's Trace	.001
	Roy's Largest Root	.001

a. Design: Intercept
Within Subjects Design: Stay + Block + Stay * Block

b. Exact statistic

Mauchly's Test of Sphericity[a]

Measure: MEASURE_1

Within Subjects Effect	Mauchly's W	Approx. Chi-Square	df	Sig.	Epsilon[b] Greenhouse-Geisser
Stay	1.000	.000	0	.	1.000
Block	1.000	.000	0	.	1.000
Stay * Block	1.000	.000	0	.	1.000

Mauchly's Test of Sphericity[a]

Measure: MEASURE_1

Within Subjects Effect	Epsilon[b] Huynh-Feldt	Lower-bound
Stay	1.000	1.000
Block	1.000	1.000
Stay * Block	1.000	1.000

Tests the null hypothesis that the error covariance matrix of the orthonormalized transformed dependent variables is proportional to an identity matrix.

a. Design: Intercept
Within Subjects Design: Stay + Block + Stay * Block

b. May be used to adjust the degrees of freedom for the averaged tests of significance. Corrected tests are displayed in the Tests of Within-Subjects Effects table.

Tests of Within-Subjects Effects

Measure: MEASURE_1

Source		Type III Sum of Squares	df	Mean Square	F
Stay	Sphericity Assumed	1250.646	1	1250.646	8.201
	Greenhouse-Geisser	1250.646	1.000	1250.646	8.201
	Huynh-Feldt	1250.646	1.000	1250.646	8.201
	Lower-bound	1250.646	1.000	1250.646	8.201
Error(Stay)	Sphericity Assumed	2897.351	19	152.492	
	Greenhouse-Geisser	2897.351	19.000	152.492	
	Huynh-Feldt	2897.351	19.000	152.492	
	Lower-bound	2897.351	19.000	152.492	
Block	Sphericity Assumed	938.165	1	938.165	17.935
	Greenhouse-Geisser	938.165	1.000	938.165	17.935
	Huynh-Feldt	938.165	1.000	938.165	17.935
	Lower-bound	938.165	1.000	938.165	17.935
Error(Block)	Sphericity Assumed	993.869	19	52.309	
	Greenhouse-Geisser	993.869	19.000	52.309	
	Huynh-Feldt	993.869	19.000	52.309	
	Lower-bound	993.869	19.000	52.309	
Stay * Block	Sphericity Assumed	1.557	1	1.557	.016
	Greenhouse-Geisser	1.557	1.000	1.557	.016
	Huynh-Feldt	1.557	1.000	1.557	.016
	Lower-bound	1.557	1.000	1.557	.016
Error(Stay*Block)	Sphericity Assumed	1817.010	19	95.632	
	Greenhouse-Geisser	1817.010	19.000	95.632	
	Huynh-Feldt	1817.010	19.000	95.632	
	Lower-bound	1817.010	19.000	95.632	

Tests of Within-Subjects Effects

Measure: MEASURE_1

Source		Sig.	Partial Eta Squared
Stay	Sphericity Assumed	.010	.302
	Greenhouse-Geisser	.010	.302
	Huynh-Feldt	.010	.302
	Lower-bound	.010	.302
Error(Stay)	Sphericity Assumed		
	Greenhouse-Geisser		
	Huynh-Feldt		
	Lower-bound		
Block	Sphericity Assumed	<,001	.486
	Greenhouse-Geisser	<,001	.486
	Huynh-Feldt	<,001	.486
	Lower-bound	<,001	.486
Error(Block)	Sphericity Assumed		
	Greenhouse-Geisser		
	Huynh-Feldt		
	Lower-bound		
Stay * Block	Sphericity Assumed	.900	.001
	Greenhouse-Geisser	.900	.001
	Huynh-Feldt	.900	.001
	Lower-bound	.900	.001
Error(Stay*Block)	Sphericity Assumed		
	Greenhouse-Geisser		
	Huynh-Feldt		
	Lower-bound		

Tests of Within-Subjects Contrasts

Measure: MEASURE_1

Source	Stay	Block	Type III Sum of Squares	df	Mean Square	F
Stay	Linear		1250.646	1	1250.646	8.201
Error(Stay)	Linear		2897.351	19	152.492	
Block		Linear	938.165	1	938.165	17.935
Error(Block)		Linear	993.869	19	52.309	
Stay * Block	Linear	Linear	1.557	1	1.557	.016
Error(Stay*Block)	Linear	Linear	1817.010	19	95.632	

Tests of Within-Subjects Contrasts

Measure: MEASURE_1

Source	Stay	Block	Sig.	Partial Eta Squared
Stay	Linear		.010	.302
Error(Stay)	Linear			
Block		Linear	<,001	.486
Error(Block)		Linear		
Stay * Block	Linear	Linear	.900	.001
Error(Stay*Block)	Linear	Linear		

Tests of Between-Subjects Effects

Measure: MEASURE_1

Transformed Variable: Average

Source	Type III Sum of Squares	df	Mean Square	F	Sig.	Partial Eta Squared
Intercept	232028.113	1	232028.113	1118.474	<,001	.983
Error	3941.561	19	207.451			

Profile Plots

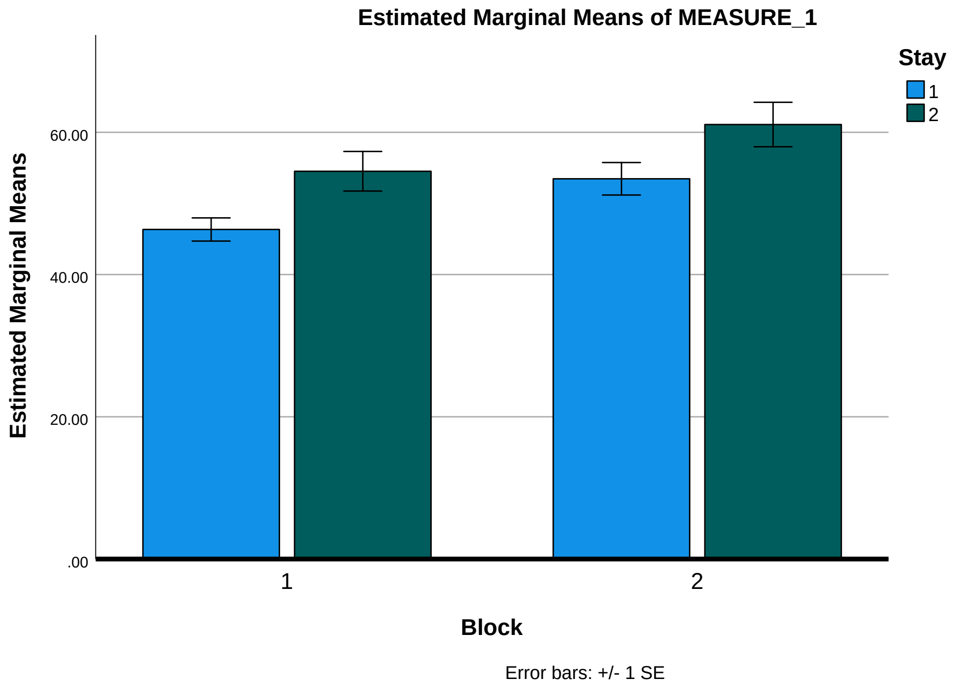

General Linear Model

Notes

Output Created		13-JUL-2022 02:22:33
Comments		
Input	Data	H:\sciebo\AA Discounting\Paper_Behavior\eNeuro\Revision_2_0\Data_and_Codes\NHST\AA_Disc_Behavior_Data_v_1_3.sav
	Active Dataset	DataSet1
	Filter	<none>
	Weight	<none>
	Split File	<none>
	N of Rows in Working Data File	25
Missing Value Handling	Definition of Missing	User-defined missing values are treated as missing.
	Cases Used	Statistics are based on all cases with valid data for all variables in the model.
Syntax		GLM Experiment_1C_Switch_Mean_Block_1 Experiment_1C_Switch_Mean_Block_2 Experiment_1C_Stay_Mean_Block_1 Experiment_1C_Stay_Mean_Block_2 /WSFACTOR=Stay 2 Polynomial Block 2 Polynomial /METHOD=SSTYPE(3) /PLOT=PROFILE (Block*Stay) TYPE=BAR ERRORBAR=SE(1) MEANREFERENCE=NO /PRINT=DESCRIPTIVE ETASQ /CRITERIA=ALPHA(.05) /WSDESIGN=Stay Block Stay*Block.
Resources	Processor Time	00:00:00,13
	Elapsed Time	00:00:00,11

Within-Subjects Factors

Measure: MEASURE_1

Stay	Block	Dependent Variable
1	1	Experiment_1C_Switch_Mean_Block_1
	2	Experiment_1C_Switch_Mean_Block_2
2	1	Experiment_1C_Stay_Mean_Block_1
	2	Experiment_1C_Stay_Mean_Block_2

Descriptive Statistics

	Mean	Std. Deviation	N
Experiment_1C_Switch_Mean_Block_1	44.2701	4.79298	21
Experiment_1C_Switch_Mean_Block_2	46.9402	8.83500	21
Experiment_1C_Stay_Mean_Block_1	44.5295	7.72333	21
Experiment_1C_Stay_Mean_Block_2	46.9033	10.76867	21

Multivariate Tests[a]

Effect		Value	F	Hypothesis df	Error df	Sig.
Stay	Pillai's Trace	.000	.003[b]	1.000	20.000	.956
	Wilks' Lambda	1.000	.003[b]	1.000	20.000	.956
	Hotelling's Trace	.000	.003[b]	1.000	20.000	.956
	Roy's Largest Root	.000	.003[b]	1.000	20.000	.956
Block	Pillai's Trace	.140	3.249[b]	1.000	20.000	.087
	Wilks' Lambda	.860	3.249[b]	1.000	20.000	.087
	Hotelling's Trace	.162	3.249[b]	1.000	20.000	.087
	Roy's Largest Root	.162	3.249[b]	1.000	20.000	.087
Stay * Block	Pillai's Trace	.000	.008[b]	1.000	20.000	.928
	Wilks' Lambda	1.000	.008[b]	1.000	20.000	.928
	Hotelling's Trace	.000	.008[b]	1.000	20.000	.928
	Roy's Largest Root	.000	.008[b]	1.000	20.000	.928

Multivariate Tests[a]

Effect		Partial Eta Squared
Stay	Pillai's Trace	.000
	Wilks' Lambda	.000
	Hotelling's Trace	.000
	Roy's Largest Root	.000
Block	Pillai's Trace	.140
	Wilks' Lambda	.140
	Hotelling's Trace	.140
	Roy's Largest Root	.140
Stay * Block	Pillai's Trace	.000
	Wilks' Lambda	.000
	Hotelling's Trace	.000
	Roy's Largest Root	.000

a. Design: Intercept
Within Subjects Design: Stay + Block + Stay * Block

b. Exact statistic

Mauchly's Test of Sphericity[a]

Measure: MEASURE_1

Within Subjects Effect	Mauchly's W	Approx. Chi-Square	df	Sig.	Epsilon[b] Greenhouse-Geisser
Stay	1.000	.000	0	.	1.000
Block	1.000	.000	0	.	1.000
Stay * Block	1.000	.000	0	.	1.000

Mauchly's Test of Sphericity[a]

Measure: MEASURE_1

Within Subjects Effect	Epsilon[b] Huynh-Feldt	Lower-bound
Stay	1.000	1.000
Block	1.000	1.000
Stay * Block	1.000	1.000

Tests the null hypothesis that the error covariance matrix of the orthonormalized transformed dependent variables is proportional to an identity matrix.

a. Design: Intercept
 Within Subjects Design: Stay + Block + Stay * Block

b. May be used to adjust the degrees of freedom for the averaged tests of significance. Corrected tests are displayed in the Tests of Within-Subjects Effects table.

Tests of Within-Subjects Effects

Measure: MEASURE_1

Source		Type III Sum of Squares	df	Mean Square	F
Stay	Sphericity Assumed	.260	1	.260	.003
	Greenhouse-Geisser	.260	1.000	.260	.003
	Huynh-Feldt	.260	1.000	.260	.003
	Lower-bound	.260	1.000	.260	.003
Error(Stay)	Sphericity Assumed	1653.449	20	82.672	
	Greenhouse-Geisser	1653.449	20.000	82.672	
	Huynh-Feldt	1653.449	20.000	82.672	
	Lower-bound	1653.449	20.000	82.672	
Block	Sphericity Assumed	133.567	1	133.567	3.249
	Greenhouse-Geisser	133.567	1.000	133.567	3.249
	Huynh-Feldt	133.567	1.000	133.567	3.249
	Lower-bound	133.567	1.000	133.567	3.249
Error(Block)	Sphericity Assumed	822.297	20	41.115	
	Greenhouse-Geisser	822.297	20.000	41.115	
	Huynh-Feldt	822.297	20.000	41.115	
	Lower-bound	822.297	20.000	41.115	
Stay * Block	Sphericity Assumed	.461	1	.461	.008
	Greenhouse-Geisser	.461	1.000	.461	.008
	Huynh-Feldt	.461	1.000	.461	.008
	Lower-bound	.461	1.000	.461	.008
Error(Stay*Block)	Sphericity Assumed	1103.469	20	55.173	
	Greenhouse-Geisser	1103.469	20.000	55.173	
	Huynh-Feldt	1103.469	20.000	55.173	
	Lower-bound	1103.469	20.000	55.173	

Tests of Within-Subjects Effects

Measure: MEASURE_1

Source		Sig.	Partial Eta Squared
Stay	Sphericity Assumed	.956	.000
	Greenhouse-Geisser	.956	.000
	Huynh-Feldt	.956	.000
	Lower-bound	.956	.000
Error(Stay)	Sphericity Assumed		
	Greenhouse-Geisser		
	Huynh-Feldt		
	Lower-bound		
Block	Sphericity Assumed	.087	.140
	Greenhouse-Geisser	.087	.140
	Huynh-Feldt	.087	.140
	Lower-bound	.087	.140
Error(Block)	Sphericity Assumed		
	Greenhouse-Geisser		
	Huynh-Feldt		
	Lower-bound		
Stay * Block	Sphericity Assumed	.928	.000
	Greenhouse-Geisser	.928	.000
	Huynh-Feldt	.928	.000
	Lower-bound	.928	.000
Error(Stay*Block)	Sphericity Assumed		
	Greenhouse-Geisser		
	Huynh-Feldt		
	Lower-bound		

Tests of Within-Subjects Contrasts

Measure: MEASURE_1

Source	Stay	Block	Type III Sum of Squares	df	Mean Square	F
Stay	Linear		.260	1	.260	.003
Error(Stay)	Linear		1653.449	20	82.672	
Block		Linear	133.567	1	133.567	3.249
Error(Block)		Linear	822.297	20	41.115	
Stay * Block	Linear	Linear	.461	1	.461	.008
Error(Stay*Block)	Linear	Linear	1103.469	20	55.173	

Tests of Within-Subjects Contrasts

Measure: MEASURE_1

Source	Stay	Block	Sig.	Partial Eta Squared
Stay	Linear		.956	.000
Error(Stay)	Linear			
Block		Linear	.087	.140
Error(Block)		Linear		
Stay * Block	Linear	Linear	.928	.000
Error(Stay*Block)	Linear	Linear		

Tests of Between-Subjects Effects

Measure: MEASURE_1

Transformed Variable: Average

Source	Type III Sum of Squares	df	Mean Square	F	Sig.	Partial Eta Squared
Intercept	175132.213	1	175132.213	1792.860	<,001	.989
Error	1953.663	20	97.683			

Profile Plots

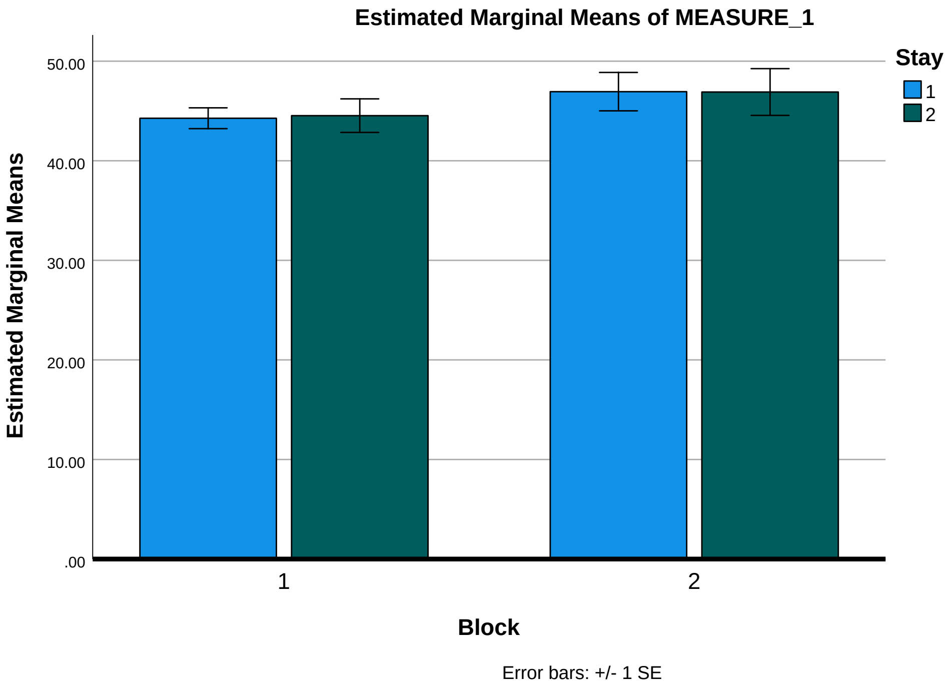

Supplement: Extended Data 1 — Data and scripts for all calculations. Folders are separated for the data extraction (Data), null hypothesis testing (NHST), and bayesian hierarchical models (Bayes). Download Extended Data 1, ZIP file [file enu-eN-NWR-0452-21-s06.zip › Data_and_Codes/NHST/OUTPUT.pdf]
