# Supplementary figures and images for: Discounting of Future Rewards and Punishments in Rats
Source: eNeuro. 2022 Nov 30;9(6):ENEURO.0452-21.2022. doi: 10.1523/ENEURO.0452-21.2022 (PMC9718352; doi:10.1523/ENEURO.0452-21.2022)

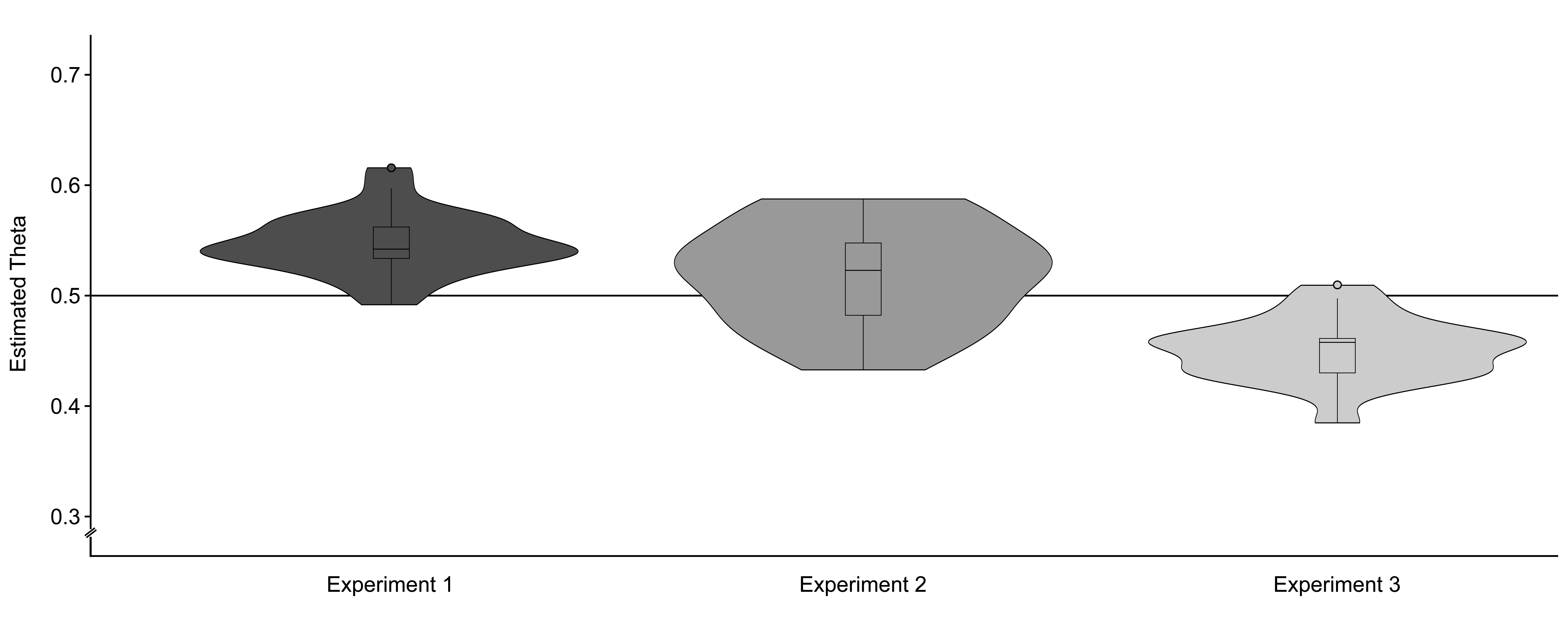

Supplement: Extended Data Figure 6-1 — Posterior θ distribution for all experiments. Displayed are violin plots where the width the density represents. Additionally, boxplots are added to the violins. Download Figure 6-1, TIF file. [file enu-eN-NWR-0452-21-s03.tif]
